# Treatment with checkpoint inhibitors in a metastatic colorectal cancer patient with molecular and immunohistochemical heterogeneity in MSI/dMMR status

**DOI:** 10.1186/s40425-019-0788-5

**Published:** 2019-11-08

**Authors:** Fotios Loupakis, Giulia Maddalena, Ilaria Depetris, Sabina Murgioni, Francesca Bergamo, Angelo Paolo Dei Tos, Massimo Rugge, Giada Munari, Andrew Nguyen, Christopher Szeto, Vittorina Zagonel, Sara Lonardi, Matteo Fassan

**Affiliations:** 1 Unit of Medical Oncology 1, Department of Oncology, Veneto Institute of Oncology, IRCCS, Via Gattamelata, 64 35128 Padua, Italy; 2 Department of Oncology, University of Padua, Padua, Italy; 3 Department of Medicine (DIMED), Surgical Pathology & Cytopathology Unit, University of Padua, Padua University Hospital, Padua, Italy; 4 Department of Pathology and Molecular Genetics, Treviso General Hospital, Treviso, Italy; 5 Nantomics, LLC, Santa Cruz, CA USA

## Abstract

**Background:**

Analysis of deficiency in DNA mismatch repair (dMMR) is currently considered a standard molecular test in all patients with colorectal cancer (CRC) for its implications in screening, prognosis and prediction of benefit from immune checkpoint inhibitors. While the molecular heterogeneity of CRC has been extensively studied in recent years, specific data on dMMR status are lacking, and its clinical consequences are unknown.

**Case presentation:**

We report the case of a metastatic CRC (mCRC) patient with immunohistochemical and molecular heterogeneity in dMMR/microsatellite instability status in the primary tumour. The patient was treated with nivolumab plus ipilimumab and achieved a deep and lasting response with clear clinical benefit. Whole-exome sequencing and RNA-seq data are reported to support the evidence for molecular heterogeneity. Re-biopsy at the time of progression ruled out the selection of MMR proficient clones as an escape mechanism. A large single-institution retrospective dataset was interrogated to further explore the real incidence of heterogeneity in its different presentations.

**Conclusions:**

The present case supports the efficacy of immune checkpoint inhibition in mCRC with heterogeneity in MMR/microsatellite instability status. Clinical issues that may arise in these rare patients are discussed in detail.

## Background

Testing for defective deficiency in DNA mismatch repair (dMMR) (or its surrogate, which is the presence of microsatellite instability, MSI) is now part of the routine diagnostic workup for patients with colorectal cancer (CRC) [1]. In fact, MSI/MMR testing is recommended in all CRC cases for Lynch syndrome screening [2];. MSI/MMR in stage II CRC identifies patients with a lower risk of recurrence and better overall survival (OS) and for whom adjuvant fluoropyrimidine monotherapy may have a questionable benefit [3, 4]. In stage IV patients, MSI/MMR is used to select candidates for immunotherapy with immune checkpoint inhibitors (ICIs) [5, 6].

Cancer is heterogeneous in nature, and this may significantly impact the personalization of patient care [7–9]. MSI/MMR status heterogeneity was recently described in gastric cancer and was associated with a lack of response to pembrolizumab [10]. In CRC, dMMR is considered an early event in the carcinogenetic process, and its heterogeneity has always been considered rather unlikely [11]. In fact, only exceptional reports of heterogeneity in the MMR pathway have been described in CRC [12–14].

Here, we describe a unique case of a metastatic CRC (mCRC) patient showing a heterogeneous MSI/MMR pattern, who was treated with ICIs and underwent an extensive molecular characterization by means of whole genome sequencing (WGS) and whole transcriptome RNA sequencing (RNA-Seq) of two MMR-discordant areas of the tumour DNA. We further discuss the relevance of MMR/MSI heterogeneity in a real world setting by re-evaluating data on MMR obtained at our centre in the last 3-year CRC series.

## Case presentation

In December 2013, a 64-year-old man was hospitalized because of worsening asthenia and abdominal pain. Past medical history included a surgically treated pT1 pN0 cM0 left clear cell renal carcinoma in 2005 and a recto-sigmoid resection for colonic low-grade tubular adenoma in 2008. Family history was positive for brain and haematologic tumours not otherwise specified in 2nd degree relatives.

During admission as an inpatient to a Unit of General Medicine of a community hospital, blood tests revealed grade 2 microcytic anaemia, and abdominal radiography showed signs of sub-occlusion. Colonoscopy revealed a right-sided neoplastic lesion. A biopsy documented an adenocarcinoma with a mucinous component. Pre-operative staging total body computed tomography (CT) including the neck, chest, abdomen and pelvis showed thickening of the right bowel wall and diffuse peritoneal nodules with maximum diameter of up to 6 cm.

**Fig. 1:**
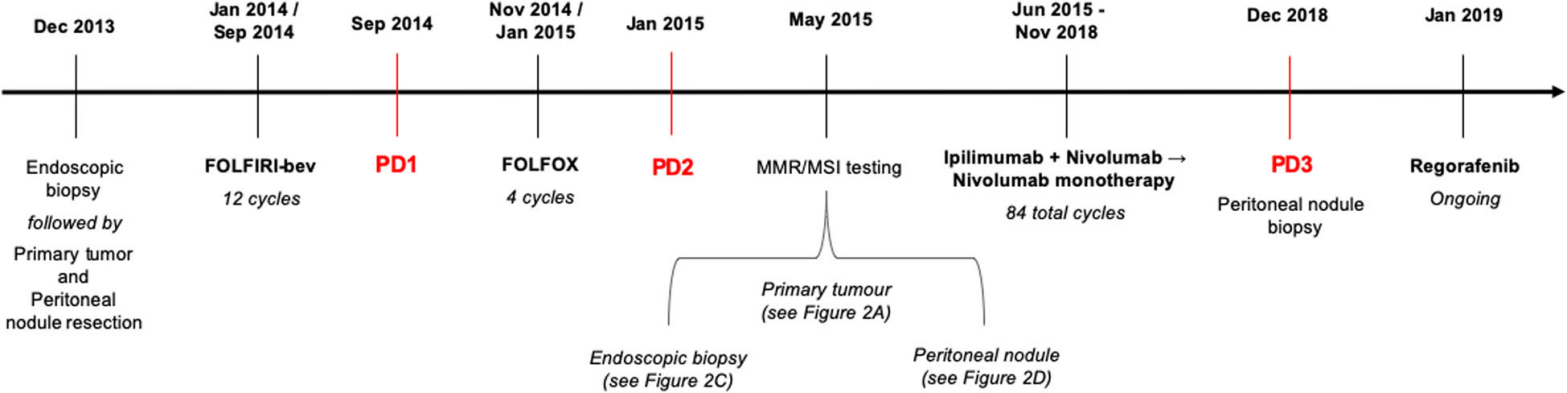
Clinical course over time, including treatments, diagnostic procedures and timing of disease progression

On December 2013, the patient underwent a palliative right hemicolectomy and diagnostic resection of a peritoneal nodule, the latter only with a diagnostic and confirmatory intent. Figure 1 summarizes the complete clinical course over time. Gross pathology examination described a 9 cm mass completely obstructing the colonic lumen. Pathological reports confirmed the diagnosis of an adenocarcinoma with the presence of heterogeneous phenotypic areas of mucinous (40% of the neoplastic area) and signet ring differentiation (pT4 pN2b [7 metastatic lymph nodes out of 10 examined], pM1c; stage IVC). Routine molecular testing showed a G12D *KRAS* mutation, whereas *NRAS*, *BRAF*, and *PIK3CA* genes showed a wild-type status (Myriapod Colon status kit; Diatech Pharmacogenetics, Jesi, Italy).

Post-operative total body CT re-evaluation confirmed the presence of large peritoneal nodules (in the right inferior, left upper and inferior abdomen), while no other lesions were detected. CEA and CA19.9 levels were within their normal range values. In February 2014, the patient was started on FOLFIRI plus bevacizumab. Tolerance was good, and partial response was documented at first re-evaluation after 4 cycles. A total of 12 cycles of therapy were delivered with regular radiologic re-evaluation every 8 weeks, confirming an initial response. In September of the same year, a CT scan showed a clear peritoneal progression of disease with the enlargement of known lesions and the appearance of new lesions.

Second-line FOLFOX was then started in November 2014. Despite a good tolerance and no treatment reductions or delays, on January 2015, a CT scan re-evaluation revealed progressive disease with dimensional increases in nodules located at the anterior abdominal wall and appearance of retroperitoneal lymph nodes. After extensive discussion of additional treatment options, the treating physicians recommended best supportive care only.

**Fig. 2:**
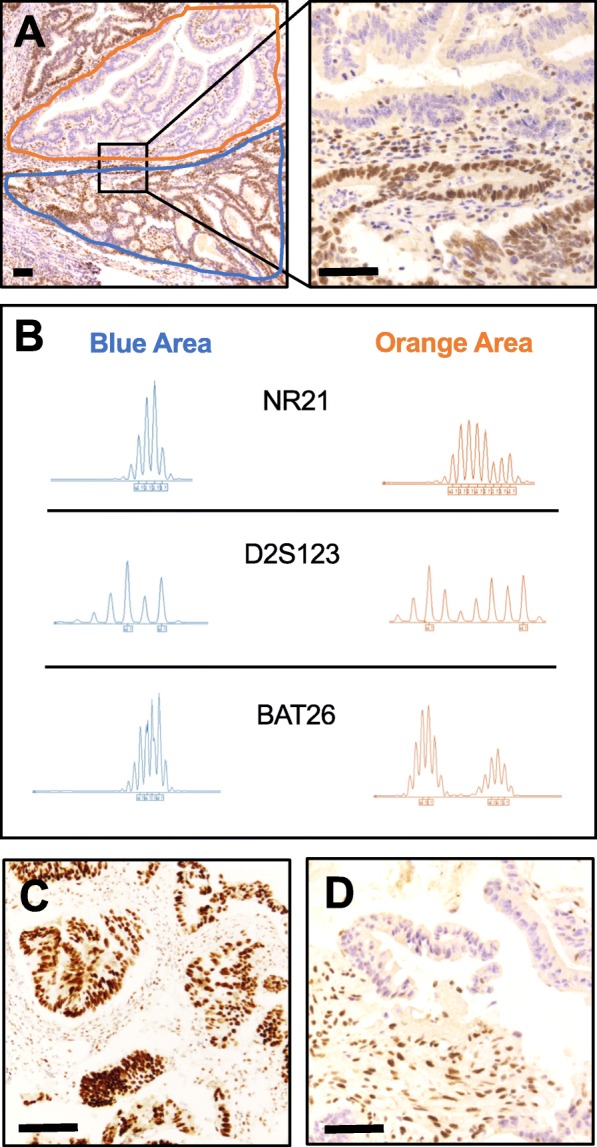
**a** Immunohistochemistry for MLH1 protein on the primary tumour showing a heterogeneous expression profile. **b** Microsatellite testing results according to different areas of the primary tumour. **c** MLH1 proficiency documented in baseline diagnostic biopsy. **d** MLH1 loss documented in synchronous peritoneal metastasis. Scale bar indicates 100 μm

The patient was referred to our Cancer Centre in May 2015. To complete the molecular evaluation of the tumour, MMR status was examined. MMR protein immunohistochemical analysis (i.e., MLH1, PMS2, MSH2, and MSH6; Dako, Glostrup, Denmark) [15] of the right-sided colonic tumour showed an unusual pattern of large areas (almost 50% of the tumour) of dMMR characterized by the complete loss of the coupled MLH1/PMS2 coexistence with areas with retained MLH1/PMS2 immunoreactivity (Fig. 2a). Based on the exceptionality of the finding, the different areas were macrodissected and tested separately for MSI (Titano kit, Diatech Pharmacogenetics, Jesi Italy), confirming previous immunohistochemistry (IHC) results (Fig. 2b).

To give a clear and comprehensive description of the case, IHC and molecular analyses were also performed on the first endoscopic biopsy and in the peritoneal metastatic nodule. The endoscopic biopsy showed a homogeneous pattern of proficiency in MMR (pMMR) (Fig. 2c), whereas the peritoneal lesion showed the complete loss of MLH1/PMS2 (Fig. 2d). Again, MSI testing confirmed the microsatellite stability (MSS) status of the biopsy and the MSI high status of the peritoneal nodule.

We further characterized the molecular landscape of this MMR heterogeneity by performing an integrated WGS and RNA-seq analysis (GPS Cancer, Nantomics, Culver City, CA) on microdissected areas of the tumour according to their different MMR/MSI statuses. Both components showed the p.G12D *KRAS* mutation and a CMS2 status according to the classification proposed by Guinney and colleagues [16]. The dMMR component presented a high tumour exonic mutational burden (TMB) with 11.0 mutations per megabase, 0.78% unstable loci (which correspond to a microsatellite instable status), and a high expression of IDO, CTLA-4 and PD-1 (Additional file 2). The pMMR component presented a low tumour exonic mutational burden (TMB) with 5.2 mutations per megabase, 5.4% unstable loci (which correspond to an MSS status), and a high expression of IDO and TIM-3 (Additional file 3). No MMR gene mutations (tumoural or germline) were identified, leading to the consideration of protein loss due to MLH1 promoter methylation.

**Fig. 3:**
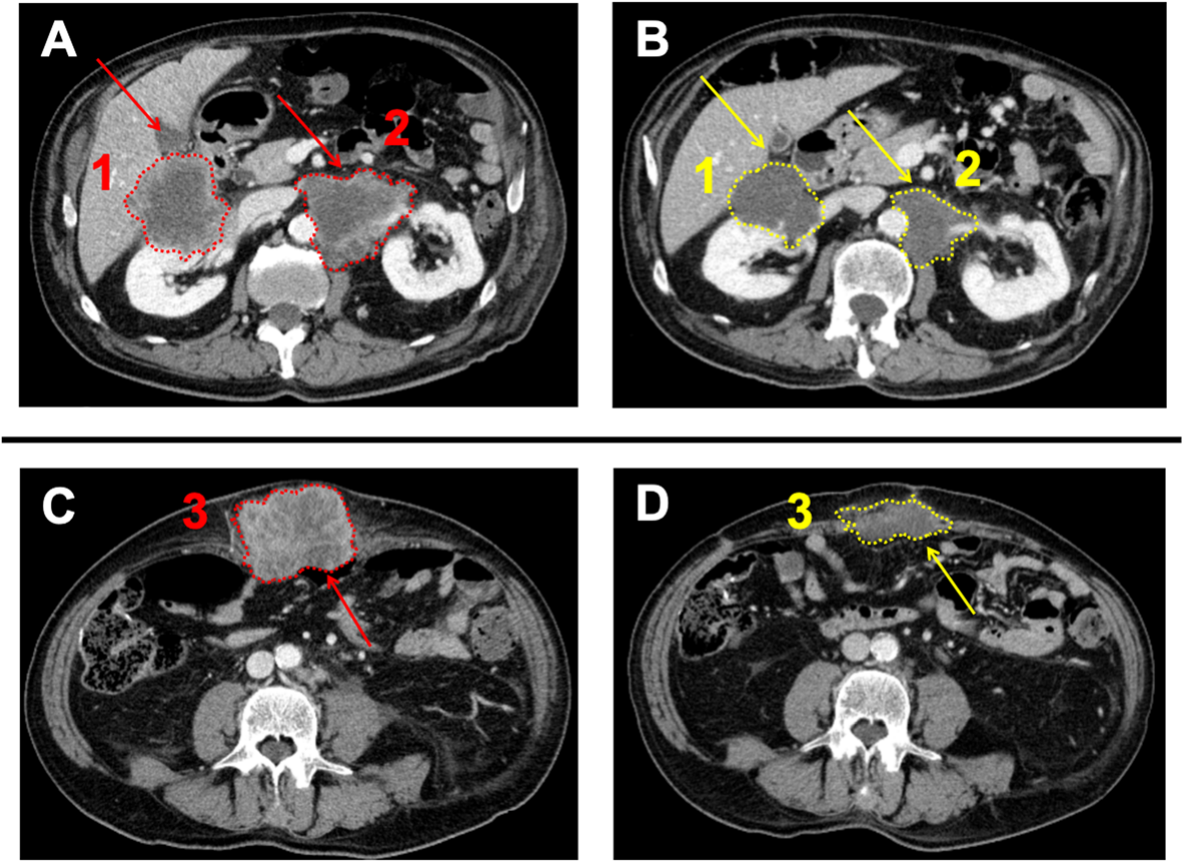
**a**-**c** Baseline CT scan images before ICI start, June 2015 (red arrows and dashed perimetral lines). Lesion A1: maximum diameter 77 mm (mm), estimated volume 1240 × 10^3^ cubic millimetres (mm^3^). Lesion A2: maximum diameter 76 mm, estimated volume 935 × 10^3^ mm^3^. Lesion C3: maximum diameter 96 mm, estimated volume 1191 × 10 [3] mm^3^. **b**-**d** Best response CT scan images, Sep 2015 (yellow arrows and dashed perimetral lines). Lesion B1: maximum diameter 48 mm, estimated volume 422 × 10^3^ mm^3^. Lesion B2: maximum diameter 42 mm, estimated volume 412 × 10 [3] mm^3^. Lesion D3: maximum diameter 79 mm, estimated volume 216 × 10^3^ mm^3^

Considering the MSI-high status of the metastatic sample, the multidisciplinary tumour board decided to start treatment with an ICI, ipilimumab 1 mg/kg plus nivolumab 3 mg/kg every 3 weeks in June 2015. After 4 cycles, chest-abdomen CT scan revealed a 32% reduction in the diameters of target lesions. i.e., partial response according to RECIST criteria 1.1 (Fig. 3). Since then, the patient was continued on nivolumab monotherapy every 2 weeks. No adverse events occurred. In November 2018, after 84 cycles and 41 months of disease control, CT showed a dimensional increase in the left antero-inferior peritoneal nodule (85 vs 69 mm), which was re-biopsied and displayed a dMMR/MSI-high status.

After disease progression during treatment with ICI, the patient was started on regorafenib. He had a good subjective tolerance, reporting no side effects, no alterations in laboratory tests and an improvement in ECOG PS (from 1 to 0). On the chest-abdomen CT re-evaluation after 8 weeks of treatment, the two bilateral inferior nodules of the peritoneum were reduced in maximum diameters (42 vs. 50 mm and 40 vs. 85 mm, respectively), and the nodules attached to the recto-sigmoid junction had signs of excavation that were compatible with the necrotic process. At the time of writing the present report, treatment with regorafenib is still ongoing.

## Discussion and conclusions

Despite the recent extensive description and characterization of the molecular heterogeneity of CRC, in everyday practice, it is considered by treating physicians as a rather homogenous disease. Reasons for this reside in a general consistency (between different areas, different metastatic lesions and over time) in the status of markers commonly tested for therapeutic purposes, such as *RAS* or *BRAF* mutations. Data on intra-tumour heterogeneity for new emerging biomarkers with therapeutic implications, such as dMMR, are limited.

Here, we describe the case of a mCRC with heterogeneous MMR/MSI status in adjacent tumour areas. The most important novel achievement was the long-lasting response with ICI followed by acquired resistance. The present case offers a unique opportunity to discuss challenges and implications for the diagnostic approach and therapeutic management of this special subgroup of CRCs.

**Fig. 4:**
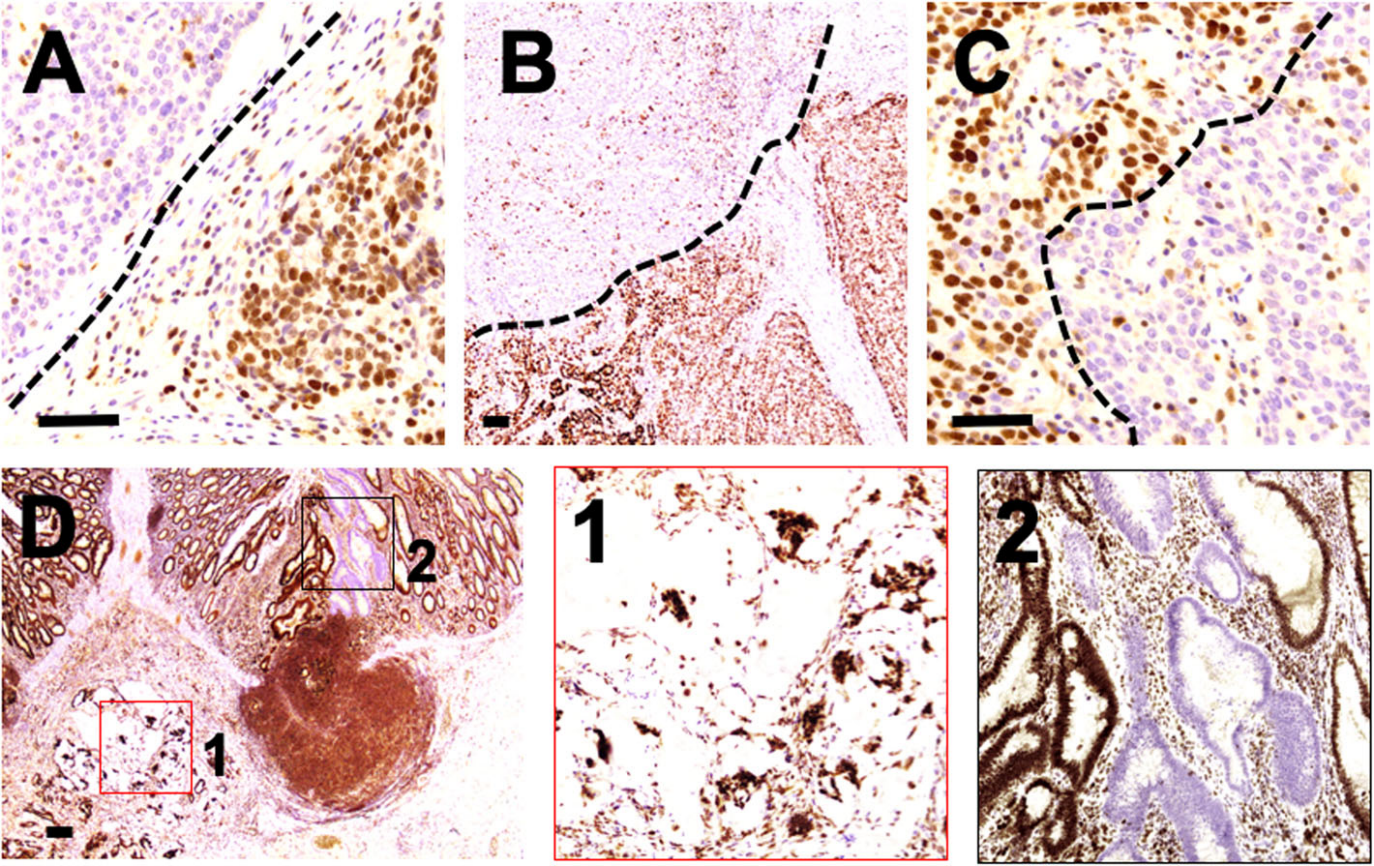
**a** Adenocarcinoma characterized by a heterogeneous MLH1 status in a tumour with complete loss of MSH2/MSH6. **b**-**c** Areas of complete loss of MSH2/MSH6 in a pMMR background. **d** A pMMR mucinous adenocarcinoma coexisting with a synchronous dMMR low-grade tubular adenoma. Scale bar indicates 100 μm

*How frequent is heterogeneity in MMR/MSI status*? To properly address this question, we reviewed our archived CRC samples over the last 3 years. A total of 1855 samples were tested for MMR protein expression by means of immunohistochemistry, and 201 (10.8%; median age 76 years, range 19–91; F/M = 0.93) showed a dMMR phenotype and 1654 a pMMR status (89.2%; median age 71 years, range 33–97; F/M = 0.62). Among the dMMR series, 13 cases showed peculiar patterns of MMR alterations (0.7%; median age 70 years, range 38–85; F/M = 0.44) (Fig. 4):

- Eight dMMR/MSI-high cases were characterized by the complete loss of the four MMR proteins in a component of the tumour and presented a second component retaining MHL1/PMS2 (*n* = 6) or MSH2/MSH6 (*n* = 2) (Fig. 4a). This finding has already been described in the context of a Lynch syndrome background [1, 17].
- MMR protein status heterogeneity, as reported in our case, was observed in two tumours, which were characterized by areas of complete loss of MSH2/MSH6 in a pMMR background (Fig. 4b-c). Of note, the microdissected dMMR areas were MSS and MSI-L by molecular testing. Due to the lack of residual material, it was not possible to further explore the genetic basis for MSH2/MSH6 protein loss and whether it was caused by MMR gene germline mutations or promoter hypermethylation (such as in rare cases with EPCAM mutations or deletions).
- Two patients with synchronous stages II/III CRCs presented different MMR statuses in the two lesions (i.e., one dMMR ad one pMMR) and, in addition, a pMMR mucinous adenocarcinoma coexisted with a synchronous dMMR (MLH1/PMS2; MSI-L) low-grade tubular adenoma (Fig. 4d1-2).

These results go beyond the aim of the present report, focused on a specific exceptional clinical case, but they show how heterogeneity - in all its forms - is a rare event in CRC. Nevertheless, it is obvious how in the era of personalized medicine, rarity should not affect the relevance for an individual patient, and the practical consequences are discussed below.

*Which could be the main clinical implications?*

A) Importance of sampling. Approximately 40% of patients with mCRC may have only a small bioptic sample available for pathological analyses at the time of initial clinical decision making [18]. Divergence between primary tumour and the biopsied tissue could be a hurdle to targeted therapies. Technical issues are extensively discussed in the literature [19]. There is a general agreement that biopsy samples could be a reliable alternative to primary tumours for *RAS* and *BRAF* mutational profiling [20]. Instead, regarding MSI status, poor data are available on sampling issues.

B) Therapeutic choice. Predictive markers of response to ICI in MSI-H/dMMR CRC are lacking and nor PD-L1 expression on tumoural cells, abudance of PD-L1 expressing tumour-associated immune cells, *BRAF* mutation status or Lynch Syndrome were predictive of benefit in the largest clinical study presented so far [21]. Despite MSI/MMR heterogeneity in the primary tumour, our patient responded to ICI treatment. Previously, Kim et al. performed a detailed molecular characterization of 61 patients with metastatic gastric cancer, 7 (11.5%) of whom had dMMR, to explore the determinant of response to pembrolizumab. Only 1 out of 7 cases with dMMR showed a lack of response and rapid progression; that tumour sample was characterized by marked geographic heterogeneity of MLH1 protein on immunohistochemical staining [10].

C) Progression to treatment. Given that mechanisms of clonal selection have already been described as escape strategies for different cancers to different targeted treatments, we initially hypothesized that MSI heterogeneity (and therefore the expansion of MSS sub-clones) could have been the major driver of acquired resistance. Notwithstanding, contrary to our expectations, molecular testing on re-biopsy at the time of progression documented a homogenous dMMR pattern. Unfortunately, the very limited tumoural content of the small biopsy performed at progression did not allow additional analyses.

*How does heterogeneity affect emerging markers of ICI efficacy?*

TMB is a validated biomarker of ICI response in metastatic melanoma, NSCLC and urothelial bladder cancer [22, 23]. In mCRC, TMB is correlated with MSI status [24] and recent data suggest a role as an independent biomarker of ICI efficacy [25]. WGS and RNA-seq analysis showed consistent findings in our case: MSI areas had high TMB, whereas MSS areas had low TMB.

Similar results were found for tumour infiltrating lymphocytes (TILs). Samples were defined as having high levels of TILs when ≥2.0 per high power field (HPF, 40x) or as having low levels of TILs when < 2.0 [26]. In all analysed specimens, we found high levels of TILs in MSI areas (consistently higher than 3.0) and no TILs in MSS areas (Additional file 1: Figure S1). Similarly, a high number of TILs was observed in the post-treatment MSI-high biopsy sample (i.e., 4.2). Galon et al. previously demonstrated this correlation between MSI-high status and immune infiltration of the tumour [27]. The number of TILs as predictive markers of response to ICI is currently under investigation by our group.

In addition to the main topics discussed above, we also report the relatively uncommon response to regorafenib. In the CORRECT trial, only 5 patients out of 500 treated with regorafenib achieved a partial response (ORR 1.0%) [28]. Therefore, the dimensional decrease and the clear necrotic effects of targeted lesions obtained by our patient were somewhat surprising. Nevertheless, it is interesting to note that expression results are in line with what was previously reported by Teufel et al. [29] regarding a greater efficacy of regorafenib in patients assigned to the consensus molecular subgroup (CMS) 2 (canonical). This evidence is preliminary, and there are no clinical implications at present.

Taken together, the information derived from the present case underlines the importance of critical and rigorous observation of patients in clinical practice, which can be crucial for collecting important data complementary to those derived from large prospective clinical trials.

## Supplementary information

**Additional file 1:** **Figure S1.** A-B-C) Tumoural areas with MMR proficiency showing no tumour infiltrating lymphocytes (TILs). D-E-F) Tumoural areas with MMR deficiency with high levels of TILs (black arrows indicating infiltrating lymphoctyes). Scale bar indicates 50 μm.

**Additional file 2.** Detailed genomic and transcriptomic analyses report for MMRp/MSS tumoral area.

**Additional file 3.** Detailed genomic and transcriptomic analyses report for MMRd/MSI tumoral area.

## Data Availability

The data analysed during the current report are available from the corresponding author on reasonable request.

## Abbreviations

CRC: Colorectal cancer
CT: Computed tomography
dMMR: deficiency in DNA mismatch repair
EPCAM: Epithelial cell adhesion molecule
ICI: Immune checkpoint inhibitors
IHC: Immunohistochemistry
MSI: Microsatellite instability
pMMR: proficiency in DNA mismatch repair
RNA-Seq: Whole-transcriptome RNA sequencing
TILs: Tumour-infiltrating lymphocytes
WGS: Whole-genome sequencing
