# Supplementary figures and images for: Treatment with checkpoint inhibitors in a metastatic colorectal cancer patient with molecular and immunohistochemical heterogeneity in MSI/dMMR status
Source: J Immunother Cancer. 2019 Nov 8;7:297. doi: 10.1186/s40425-019-0788-5 (PMC6842181; doi:10.1186/s40425-019-0788-5)

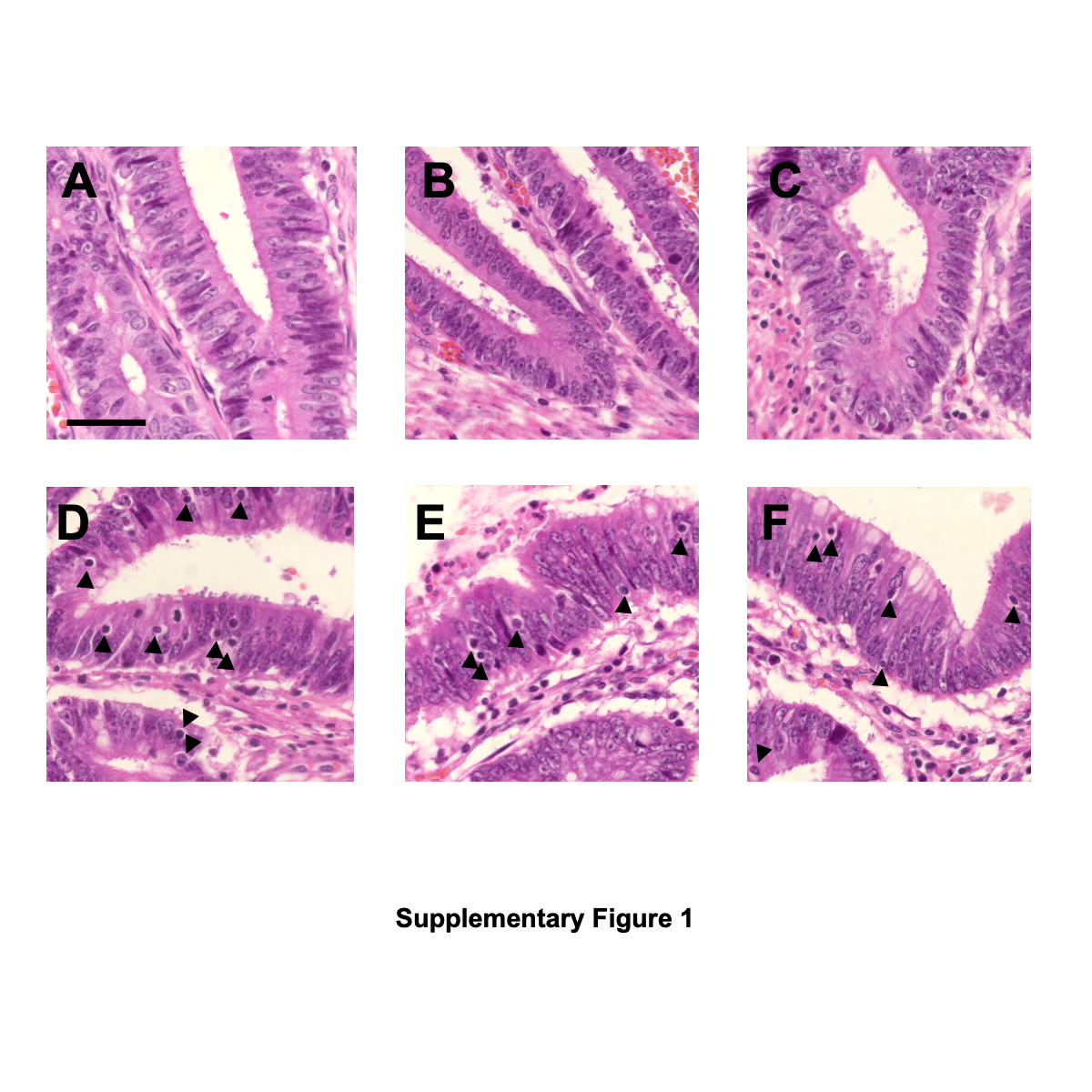

Supplement: Supplementary file 1 — Additional file 1: Figure S1. A-B-C) Tumoural areas with MMR proficiency showing no tumour infiltrating lymphocytes (TILs). D-E-F) Tumoural areas with MMR deficiency with high levels of TILs (black arrows indicating infiltrating lymphoctyes). Scale bar indicates 50 μm. [file 40425_2019_788_MOESM1_ESM.tiff]
