# Supplementary material for: Treatment with checkpoint inhibitors in a metastatic colorectal cancer patient with molecular and immunohistochemical heterogeneity in MSI/dMMR status
Source: J Immunother Cancer. 2019 Nov 8;7:297. doi: 10.1186/s40425-019-0788-5 (PMC6842181; doi:10.1186/s40425-019-0788-5)
Supplement: Supplementary file 2 — Additional file 2. Detailed genomic and transcriptomic analyses report for MMRp/MSS tumoral area. [file 40425_2019_788_MOESM2_ESM.pdf]

Research Report

Report ID 124_PA_PD

Genomic and Transcriptomic Analyses	Results
Tumor Exonic Mutational Burden	**HIGH** (233 non-synonymous / 11.0 per Mb) (pg. 4)
Germline Cancer Predisposition Screening	**NO DELETERIOUS, TRUNCATING VARIANTS DETECTED** (pg. 7)
Microsatellite Status	**UNSTABLE** (MSI+) (pg. 7)
Disruptive germline alterations identified in	ITPA, SV2C, CYP3A5, UGT1A1, FCAMR (pg. 9)
Purity / Ploidy / % LOH	**55%** / **2.2n** / **10.2%** (pg. 11)
Site of Origin Prediction	**Colorectal** (pg. 12)
Inferred Hormone Receptor Status	**ER- / PR- / Her2-** (pg. 13)

More findings can be found in the main report below.

Potentially Effective Drugs	Based Upon
✓ **Nivolumab, Pembrolizumab** (pg. 2)	Microsatellite Instability (DNA+)

Sensitivity biomarkers curated from sources outside of FDA-approved indications were detected for: **Irinotecan**, **Topotecan**.

Potentially Ineffective Drugs	Based Upon
✗ **Cetuximab, Panitumumab** (pg. 2)	*KRAS* p.G12D (DNA+, RNA+)

Resistance biomarkers curated from sources outside of FDA-approved indications/contraindications were detected for: **Erlotinib**, **Erlotinib or Gefitinib**, **Gefitinib**.

Available Samples	Tumor DNA + RNA, Matched-Normal DNA (Provenance Confirmed)
Variants Reported	Somatic & Germline SNVs, MNVs, Insertions & Deletions (+ RNA), Amplifications

MSI+ Curated Findings

**MSI+**<br>Evidence	Genomics<br>5.4% Sites Unstable★	Transcriptomics<br>N/A

Indications

✓ **Pembrolizumab** is indicated for the treatment of adult and pediatric patients with unresectable or metastatic, microsatellite instability-high (MSI-H) or mismatch repair deficient:

1. colorectal cancer that has progressed following treatment with a fluoropyrimidine, oxaliplatin, and irinotecan.
2. solid tumors that have progressed following prior treatment and who have no satisfactory alternative treatment options.

★ MSI-H, *Source:* KEYTRUDA (pembrolizumab) FDA label (June 2018), 448 active clinical trials

✓ **Nivolumab** is indicated for the treatment of adult and pediatric patients 12 years and older with microsatellite instability-high (MSI-H) or mismatch repair deficient (dMMR) metastatic colorectal cancer (CRC) that has progressed following treatment with a fluoropyrimidine, oxaliplatin, and irinotecan.

★ MSI-H, *Source:* OPDIVO (nivolumab) FDA label (Apr. 2018), 365 active clinical trials

Limitations

? **Pembrolizumab** efficacy and safety have not been established for pediatric patients with MSI-H central nervous system cancers.

★ MSI-H, *Source:* KEYTRUDA (pembrolizumab) FDA label (June 2018)

KRAS Curated Findings

**KRAS**<br>Evidence	Genomics<br>Normal Copy Number (2.2x)<br>p.G12D (50% AF)★	Transcriptomics<br>Expressed (17.7 TPM)<br>p.G12D (34% AF)★

Not Indicated

✗ **Cetuximab** is not indicated for treatment of K-Ras mutation-positive colorectal cancer.

★ KRAS mutation in codons 12, 13, 59, 61, 117 or 146, *Source:* ERBITUX (cetuximab) FDA Label (Oct. 2016)

✗ **Panitumumab** is not indicated for the treatment of patients with RAS-mutant metastatic colorectal cancer or for whom RAS mutation status is unknown.

★ KRAS mutation in codons 12, 13, 59, 61, 117 or 146, *Source:* VECTIBIX (panitumumab) FDA label (June 2017)

✗ **Cetuximab** is not indicated for the treatment of patients with colorectal cancer that harbor somatic mutations in exon 2 (codons 12 and 13), exon 3 (codons 59 and 61), and exon 4 (codons 117 and 146) of either K-Ras or N-Ras.

★ KRAS G12X, *Source:* ERBITUX (cetuximab) FDA Label (Oct. 2016)

Therapy / Biomarker Comparisons

- Adenocarinoma non-small cell lung cancer patients treated with **Erlotinib or Gefitinib** were associated with worse outcomes if they had *KRAS mutation in codon 12 or 13* compared to *KRAS wildtype at codon 12 and 13* as measured by: Overall Survival (OS) and Progression Free Survival (PFS)

  ★ KRAS mutation in codon 12 or 13 vs. KRAS wildtype at codon 12 and 13, *Source:* PubMed 19794967

Drug Resistance

▼ Confers resistance to **Cetuximab**.

★ KRAS mutation in codon 12 or 13, *Source:* PubMed 25623215

▼ Associated with resistance to **Cetuximab** (1, 2, 3, 4, 5, 6), **Panitumumab** (10, 2, 7, 8, 9), **Erlotinib or Gefitinib** (11), **Erlotinib** (12), **Gefitinib** (12).

★ KRAS G12D, *Sources:* (1) PubMed 16618717, (2) PubMed 17363584, (3) PubMed 26888827, (4) PubMed 18202412, (5) PubMed 17998284, (6) PubMed 25623215, (7) PubMed 23041588, (8) PubMed 18316791, (9) PubMed 24025413, (10) PubMed 18621636, (11) PubMed 19794967, (12) PubMed 15696205

MGMT Curated Findings

**MGMT** Evidence	Genomics	Transcriptomics
	Normal Copy Number (1.9x)	Over-Expressed (24.7 TPM)★

Therapy / Biomarker Comparisons

- Glioblastoma patients treated with **Temozolomide** were associated with worse outcomes if they had *high MGMT expression* compared to *low MGMT expression* as measured by: Objective Response Rate (ORR), Overall Survival (OS), and Progression Free Survival (PFS)
  ★ high MGMT expression vs. low MGMT expression, *Source:* PubMed 17442989

TOP1 Curated Findings

**TOP1** Evidence	Genomics	Transcriptomics
	Normal Copy Number (2.8x)	Over-Expressed (114.9 TPM)★

Drug Sensitivity

▲ Inhibited by **Irinotecan** (1, 2, 3), **Topotecan** (4, 5).
★ expresses TOP1, *Sources:* (1) PubMed 1651156, (2) CAMPTOSAR (Irinotecan) FDA label (Dec. 2014), (3) ONIVYDE (irinotecan liposome injection) FDA label (Oct. 2015), (4) PubMed 7743509, (5) HYCAMTIN (topotecan) FDA label (June 2015)

Molecular Oncology Findings

About this analysis: Alterations to known oncogenes, known tumor suppressors, and/or potentially treatable genes are gathered by this analysis. Small somatic variants presented here must be classified as "Pathogenic" or "Likely Pathogenic" according to the method described in the *Additional Mutation Analysis* section below. Treatable genes are those identified in a targeted treatment above and are not necessarily known oncogenes or tumor suppressors. *See Methods section for more details.*

A total of 619,944 somatic variants were identified in this patient's tumor, including 233 non-synonymous variants, for an estimated exonic mutation rate of 11.0 mutations per megabase (Mb).

Tumor sample's exonic mutation rate (red line) compared to 5,013 tumor samples

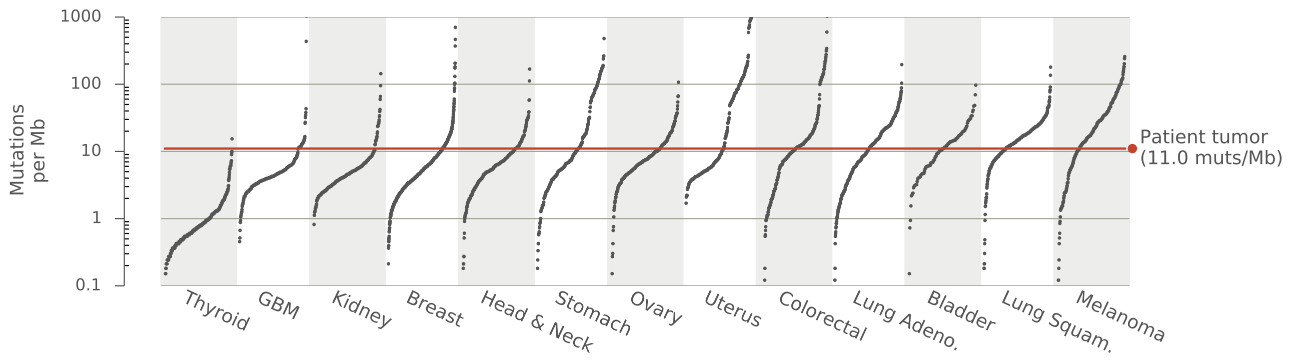

Tumor exonic mutational burden is **HIGH** according to the number of non-synonymous mutations detected in the tumor sample, where mutational burden is classified as HIGH in tumors with 200 or more non-synonymous mutations and LOW in tumors with fewer than 200 non-synonymous mutations. A somatic non-synonymous mutation burden exceeding 200 mutations has been shown to be associated with improved responsiveness to anti-PD-1 therapy (Rizvi et al (15) Science 248: 124). The increased somatic mutational burden may be predictive of response to immune checkpoint inhibitors.

Of the 102 oncogenes and 137 tumor suppressors analyzed here, 5 small variants (predicted to be Pathogenic, Likely Pathogenic, and/or treatable) and 3 copy number alterations were detected. These variants are presented in the tables below, with oncogenes labeled in *RED* and tumor suppressors labeled in *BLUE*.

Small variants in known cancer and/or treatable genes

Biomarker	Alt. / Total	RNA Alt. / Total	Details
TP53 p.V217Wfs*31 (DNA+, RNA+)	15 / 74	7 / 36	Pathogenic, Tumor Suppressor
KRAS p.G12D (DNA+, RNA+)	6 / 22	28 / 82	Pathogenic, Oncogene
APC p.E1306* (DNA+, RNA+)	13 / 22	27 / 38	Pathogenic, Tumor Suppressor
MAP2K4 Loss (DNA+)			Tumor Suppressor
FGFR4 p.A447T (DNA+, RNA+)	6 / 101	10 / 243	Likely Pathogenic, Oncogene
ABL1 p.A621T (DNA+, RNA+)	6 / 119	4 / 516	Likely Pathogenic, Oncogene

Copy number alterations in known cancer and/or treatable genes

Biomarker	Copy Number	TPM	Details
APC Loss (DNA+)	0.17	15.89	Tumor Suppressor
SETBP1 Loss (DNA+)	0.46	17.49	Oncogene

Protein Analysis

About this analysis: Using mass spectrometry, a set of 22 clinical protein markers are quantified and used to identify treatments with *Likely*, *Unlikely*, and *Uncertain* benefit. When applicable, an 4 additional protein markers are quantified by the LungAdenoPlex assay to determine cellular origin of lung tumors, the P16 protein is measured to determine the likelihood of HPV infection in head & neck cancers, and the KRAS protein is measured for predicting poor prognosis in gastroesophageal and endometrial cancers. *See Methods section for more details.*

Protein measurements are not available for this sample.

Genes Associated with Chemotherapy Response

About this analysis: Expression levels for 8 genes associated with differential chemotherapy response are presented here. An empirically-derived cutoff for each gene is used to determine if its expression level is *High*, which may indicate the tumor's sensitivity (or resistance) to the associated drugs. *See Methods section for more details.*

The expression data for genes associated with chemotherapy response are summarized in the tables below.

Sensitivity-associated genes

Gene	Protein	Associated Drug(s)	Expression Status	TPM	Cutoff (TPM)	Median (Clinical, TPM)
TOP1	TOPO1	**Irinotecan**, **Topotecan**	**High**	114.92	101.01	79.41
TYMP	TYMP	**5-FU**, **Capecitabine**	-	163.88	224.13	163.46
SLC29A1	hENT1	**Gemcitabine**	-	41.94	108.62	71.72
FOLR1	FR-alpha	**Pemetrexed**	-	1.04	38.88	3.44
TOP2A	TOPO2A	**Doxorubicin**, **Epirubicin**, **Etoposide**	-	59.06	109.23	39.68

Resistance-associated genes

Gene	Protein	Associated Drug(s)	Expression Status	TPM	Cutoff (TPM)	Median (Clinical, TPM)
RRM1	RRM1	**Gemcitabine**	-	31.79	94.54	38.91
TUBB3	TUBB3	**Paclitaxel**, **Docitaxel**	-	25.59	93.72	43.38
MGMT	MGMT	**Temozolomide**	**High**	24.66	12.25	30.86

Additional Mutation Analysis

About this analysis: The pathogenicity of somatic small mutations is predicted using a heuristics-based procedure oriented towards cancer. Information at the gene-level (oncogene or tumor suppressor, driver of tumorigenesis, etc.) and information specific to the mutation (activating or disruptive, conservation score, location within recurrently-mutated hotspot, etc.) is utilized. While all somatic small variants are analyzed by this procedure, variants to Known Oncogenes and Tumor Suppressors classified as "Pathogenic" or "Likely Pathogenic" are not presented in detail here, as they are reported in *Molecular Oncology Findings* analysis above. *See Methods section for more details.*

All somatic variants identified in this patient sample were categorized according to the each variant's annotation and supporting data as described in the Methods. A summary of mutations broken down by variant class and pathogenicity category is provided below. Please note that mutations listed in the *Molecular Oncology Findings* section are included in the summary table below.

Summary of variants by class and pathogenicity

Category	Total	Nonsense	Missense	Frame Shift	In-Frame	Splice Site	Silent*
Pathogenic	3	1	1	1	-	-	-
Likely Path.	5	-	5	-	-	-	-
Unkn. Sig.	31	8	18	5	-	-	-
Likely Benign	4	-	3	-	1	-	-
Benign	610,012	-	181	-	5	1	609,825
Common	9,889	-	3	-	-	-	9,886

* Silent variant class includes synonymous variants, variants in non-coding RNAs, and variants in intronic or intergenic regions of the genome.

The table below lists up to 20 variants not reported above in the *Molecular Oncology Findings* section, ranked according to their predicted pathogenicity. The *Details* column lists classifications used in determining the variant's *Category*. All remaining variants predicted as "Pathogenic", "Likely Pathogenic", or "Unknown Significance" are available in the appendix. Clonality status was determined using a purity estimate of 55% and a ploidy estimate of 2.1n.

Top additional mutations ranked by pathogenicity

Category	Gene	Class	Variant	Alt. / Total	RNA Alt. / Total	Details
Likely Path.	*ZNF479*	Missense	p.R295K	6 / 63	3 / 5	RNA+, Putative Oncogene, Subclonal, In COSMIC (N=10)
Likely Path.	*SRRM2*	Missense	p.E1558G	5 / 82	53 / 2233	RNA+, Putative Driver Gene, CASM High, Subclonal
Likely Path.	*ZNF479*	Missense	p.T382K	4 / 47	2 / 2	RNA+, Putative Oncogene, Subclonal
Unkn. Sig.	*CDC27*	Missense	p.L275V	6 / 47	22 / 93	RNA+, Putative Tumor Suppressor, Subclonal, In COSMIC (N=4)
Unkn. Sig.	*ZNF91*	Missense	p.K829E	5 / 89	443 / 1114	RNA+, Driver Gene, Subclonal, In COSMIC (N=2)
Unkn. Sig.	*SYNE1*	Missense	p.R8064C	29 / 121	0 / 64	RNA-, Driver Gene, Clonal, In COSMIC (N=1)
Unkn. Sig.	*CFH*	Missense	p.V149L	5 / 23	0 / 89	RNA-, Driver Gene, In COSMIC (N=1)
Unkn. Sig.	*CDC27*	Missense	p.N260H	4 / 43	14 / 70	RNA+, Putative Tumor Suppressor, Subclonal, In COSMIC (N=1)
Unkn. Sig.	*ZC3H18*	Missense	p.R479H	48 / 115	198 / 315	RNA+, Putative Tumor Suppressor, Clonal
Unkn. Sig.	*OR4N4*	Missense	p.P58H	21 / 41	0 / 0	RNA-, Hotspot, Clonal
Unkn. Sig.	*KPRP*	Missense	p.P446Q	14 / 87	0 / 0	RNA-, CASM High, Subclonal
Unkn. Sig.	*PASD1*	Nonsense	p.Q766*	13 / 66	0 / 0	RNA-, Not NMD, Clonal
Unkn. Sig.	*CFAP61*	Nonsense	p.R283*	12 / 44	0 / 0	RNA-, NMD
Unkn. Sig.	*RFESD*	Missense	p.R109L	10 / 28	2 / 6	RNA+, CASM High, Clonal
Unkn. Sig.	*NBAS*	Nonsense	p.R57*	9 / 31	19 / 95	RNA+, NMD
Unkn. Sig.	*ATRNL1*	Missense	p.C1014F	9 / 32	0 / 6	RNA-, CASM High
Unkn. Sig.	*LEPR*	Missense	p.L939F	7 / 48	7490 / 7969	RNA+, Driver Gene, Subclonal
Unkn. Sig.	*ZNF493*	Missense	p.E681G	6 / 78	86 / 209	RNA+, CASM High, Subclonal
Unkn. Sig.	*CCDC120*	Missense	p.A341V	6 / 70	30 / 203	RNA+, Putative Tumor Suppressor, Subclonal
Unkn. Sig.	*MAST2*	Missense	p.P12S	5 / 55	36 / 896	RNA+, Driver Gene, Subclonal

RNA Rescue: COSMIC SNVs in Cancer Genes

About this analysis: Finds support for COSMIC single nucleotide variants in known oncogenes and tumor suppressors in RNA sequencing data that were not detected in the DNA sequencing data. *See Methods section for more details.*

A total of 1 variant were rescued from the RNA sequencing data by this analysis, up to 20 of these variants are summarized in the table below.

Summary of variants found by RNA rescue

Gene	Variant	Normal Alt / Total	Tumor Alt / Total	RNA Alt / Total	Details
PIK3CA	`c.1913T>C; p.V638A`	0 / 35	0 / 32	8 / 32	RNA Rescued, # COSMIC = 4

Secondary Screening for Cancer Predisposition

About this analysis: Disruptive germline alterations (nonsense single nucleotide variants or frame-shifting insertions & deletions) in a panel of 22 genes implicated in increased risk of developing cancer are presented here. Genes in this list must demonstrate autosomal dominant or semi-dominant inheritance of disruptive alterations related to cancer predisposition. Predictions of nonsense-mediated decay (NMD) are provided for all reported variants. *See Methods section for more details.*

No relevant variants were identified in cancer predisposition genes in the patient's germline sequencing data.

Microsatellite Instability

About this analysis: Up to 2,848 microsatellite repeats are analyzed in tumor and normal sequencing datasets to estimate the degree of microsatellite instability (MSI) exhibited by the tumor. In addition, disruptive alterations detected within the coding regions of 4 DNA repair genes are collected here to help identify potential genetic causes of any observed instability in the tumor genome. *See Methods section for more details.*

Out of the 1,673 microsatellites analyzed in the tumor with sufficient coverage, 109 were deemed unstable. The tumor demonstrated a difference of 5.44% unstable loci relative to the normal sample, characteristic of a genome exhibiting microsatellite instability (MSI+ > 2.50%). The summary of the analysis can be found in the table below.

Summary of microsatellite analysis on patient's samples.

Sample	# Loci	% Unstable	Differential	Microsatellite Status
Tumor	1,673	6.52	5.44	MSI+
Normal	747	1.07		

A summary of disruptive alterations occuring in 4 DNA repair genes (MLH1, MSH2, MSH6, PMS2) is provided in the table below. Within these genes, the analysis detected 4 alterations in the patient's sequencing data that may influence the stability of microsatellite repeats in the tumor genome.

Disruptive alterations detected in DNA repair genes.

Gene	Class	Variant
MLH1	Expression	Normal (23.55 TPM)
MSH2	Expression	Normal (28.17 TPM)
MSH6	Expression	Over-expressed (38.78 TPM)
PMS2	Expression	Normal (24.35 TPM)

Copy number analysis

About this analysis: A survey of relative coverage and allelic imbalances is performed across the whole genome to identify regions exhibiting altered copy number. Only regions harboring at least one gene of interested are reported, up to a maximum of 10 regions with the highest and lowest relative coverage estimates. Reported regions must be at least 10kb in size. *See Methods section for more details.*

A total of 7,328 regions were identified according to the methods described below. The tables below list up to 10 regions of the tumor genome that harbor at least one gene of interest with the highest and lowest copy number copy number estimates. Copy number was computed by converting relative coverage estimates with a purity estimate of 55% and a ploidy estimate of 2.1n.

Top ranked regions harboring genes of interest

Status	Copy Number	Genes of Interest
Moderate Amp.	5.8 +/- 3.0	*TEX11*
Moderate Amp.	5.7 +/- 3.1	*ATRX*
Normal	5.5 +/- 3.0	*BRWD3*
Moderate Amp.	5.2 +/- 2.2	*UPF3B*
Normal	5.1 +/- 2.7	*STAG2*
Moderate Amp.	5.1 +/- 2.5	*MED12*, *ZMYM3*, *NONO*, *TAF1*
Normal	4.9 +/- 2.6	*RBMX*
Normal	4.9 +/- 2.7	*KDM6A*
Normal	4.9 +/- 3.3	*GPR174*
Normal	4.7 +/- 3.4	*AMER1*

Bottom ranked regions harboring genes of interest

Status	Copy Number	Genes of Interest
Loss	0.5 +/- 1.0	*SETBP1*
Normal	0.9 +/- 1.2	*PIK3R1*
Normal	0.9 +/- 1.3	*PLK2*
Normal	1.0 +/- 1.0	*MAP3K1*
Normal	1.0 +/- 1.2	*MBD1*, *SMAD4*
Normal	1.1 +/- 1.1	*CDH7*
Normal	1.1 +/- 1.1	*NEDD4L*
Normal	1.1 +/- 1.3	*TRIM23*, *ERBB2IP*
Normal	1.2 +/- 1.0	*SMAD2*
Normal	1.3 +/- 1.3	*MITF*

The figure below shows the estimated relative coverage (gray, top plot) and majority allele fraction (red, bottom plot) across the 22 autosomal chromosomes, showing the extent of copy number variation in this tumor. The area in red on the majority allele fraction plot represents the fraction of all of observed reads with the majority allele in that region of the tumor genome. Only heterozygous sites in the matched-normal sample are utilized in this plot. For example, a region with majority allele fraction of 1.0 means all heterozygous sites in that genomic region of the matched-normal sample are now homozygous for the majority allele in the tumor genome (i.e. possible loss of heterozygosity). Note that maximal majority allele fractions are correlated with tumor purity.

Tumor copy number and majority allele fraction

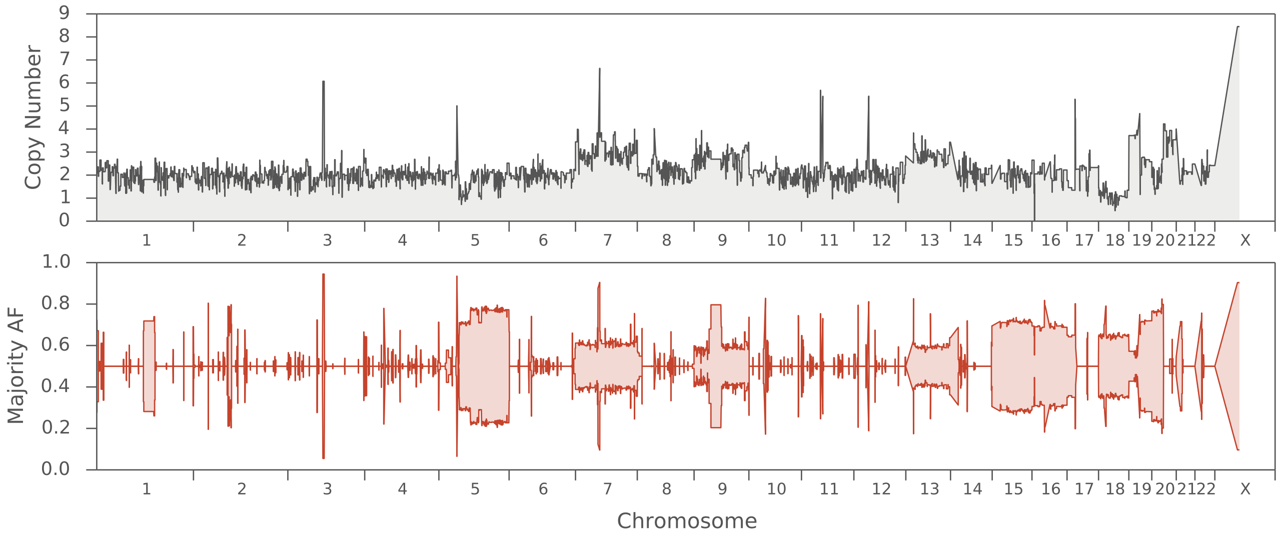

Pharmacogenomics Screening

About this analysis: Disruptive germline alterations (nonsense single nucleotide variants or frame-shifting insertions & deletions) in a panel of 22 genes implicated in increased risk of toxicity in response to anti-cancer therapies are presented here. *See Methods section for more details.*

Based on an analysis of patient's germline data, a total of 8 variants implicated in altered toxicity of anti-cancer drugs were present in 5 genes.

Toxicity related findings linked to variants detected in the patient.

Genotype	Finding	Recommendation Strength
CYP3A5 *1/*3	*Intermediate Metabolizer of* **Tacrolimus**<br>Risk of decreased dose-adjusted trough concentrations, possibly delaying achievement of target blood concentrations. Increase dose to achieve therapeutic drug concentrations. *Source: CPIC guideline*	Strong
UGT1A1 *28/*28	**Belinostat**<br>UGT1A1 *28/*28 confers increased risk in the development of belinostat-induced toxicities. Patients with UGT1A1 *28/*28 receiving belinostat should have a starting dose reduced to 750 mg/m2 to minimize dose limiting toxicities. *Source: FDA Label*	N/A
	**Irinotecan**<br>UGT1A1 *28/*28 confers increased risk in the development of neutropenia following treatment with irinotecan. Patients with UGT1A1 *28/*28 receiving irinotecan should consider reducing starting dose by one level. *Source: FDA Label*	N/A
	**Nilotinib**<br>UGT1A1 *28/*28 confers increased risk in the development of hyperbilirubinemia following treatment with nilotinib. *Source: FDA Label*	N/A
	**Pazopanib**<br>UGT1A1 *28/*28 confers increased risk in the development of hyperbilirubinemia following treatment with pazopanib. *Source: FDA Label*	N/A

The *1 allele indicates no variant (wild-type)

Pharmacogenomic associations described in the primary literature

Variant	Zygosity	Drug	Toxicity Info
FCAMR rs1856746	Homozygous	**Taxane**	FCAMR rs1856746 is positively correlated with increased risk in the development of taxane-induced peripheral neuropathy (TIPN). *Source: Pubmed 26138065*
ITPA rs1127354	Heterozygous	**Mercaptopurine**	ITPA rs1127354 is associated with increased risk in the development of mercaptopurine-induced neutropenia. *Source: Pubmed 18685564*
SV2C rs6453204	Homozygous	**Bevacizumab**	SV2C rs6453204 is positively correlated with increased risk in the development of bevacizumab-induced hypertension. *Source: Pubmed 25117820*

No somatic variants detected in patient.

Status of HRD-Related Genes

About this analysis: In tumors with large contributions of the BRCA1/2 mutational signatures, this analysis attempts to find genetic factors that may explain homologous recombination defiency (HRD), such as disruptive somatic and/or germline variants, copy number alterations, and expression status of genes related to HRD (*BRCA1*, *BRCA2*, *PALB2*, *RAD51C*). *See Methods section for more details.*

Based on the high percentage of mutations attributed to the BRCA1/2 mutational signature (16.3%), it is likely that this tumor is homologous recombination deficient (HRD).

The patient's sequencing data were analyzed for alterations to genes related to homologous recombination deficiency. The summary of findings is provided in the table below.

Status of HRD-related genes

Gene	Copy Number	Allelic State (A, B)	Exp. Status (TPM)	Variant(s)
BRCA1	Normal (2.9x)	Gain (2, 1)	Over-expressed (20.09 TPM)	-
BRCA2	Normal (2.8x)	Gain (2, 1)	Over-expressed (7.81 TPM)	-
PALB2	Normal (2.1x)	CN-LOH (2, 0)	Over-expressed (15.68 TPM)	-
RAD51C	Normal (2.9x)	Normal (1, 1)	Normal (12.01 TPM)	-

Cytogenetic Analysis

Based on the analysis described in the *Methods*, the tumor sample's copy number data suggests it is composed of approximately 55% tumor and 45% normal tissue. The table below lists up to 10 regions of the tumor genome exhibiting an altered and approximately integral allelic state along with their overlapping cytoband. Copy number segments with normal allelic states (1, 1) or those that do not approximate integral allelic states (as described in the *Methods*) are not included in the table. The tumor genome has an estimated ploidy of 2.2n and approximately 88% of the genome is in a clonal allelic state. A figure displaying the allelic state diagrams and copy number and majority allele fraction chromosomal plots for the 22 autosomal chromosomes can be found in the Appendix.

Altered allelic copy number states for regions larger than 2.5Mb, assuming tumor sample purity is 55%

Location	(Major, Minor)	Size (Mb)	Cytoband(s)	State
5:51,836,002-77,957,001	(1, 0)	26.12	5q11.2 - 5q14.1	LOH
5:82,247,002-102,715,001	(2, 0)	20.47	5q14.2 - 5q21.1	CN-LOH
5:102,715,002-109,453,001	(1, 0)	6.74	5q21.1 - 5q21.3	LOH
5:109,702,002-175,698,001	(2, 0)	66.00	5q22.1 - 5q35.2	CN-LOH
7:7,618,002-138,130,001	(2, 1)	130.51	7p21.3 - 7q33	Moderate Amp.
7:142,794,002-150,186,001	(2, 1)	7.39	7q34 - 7q36.1	Moderate Amp.
7:152,446,002-156,489,001	(2, 1)	4.04	7q36.1 - 7q36.3	Moderate Amp.
9:24,646,002-108,485,001	(2, 1)	83.84	9p21.3 - 9q31.2	Moderate Amp.
9:135,951,002-138,955,001	(2, 1)	3.00	9q34.2 - 9q34.3	Moderate Amp.
13:23,160,002-112,930,001	(2, 1)	89.77	13q12.11 - 13q34	Moderate Amp.

Cytogenetic plot of inferred integral copy number

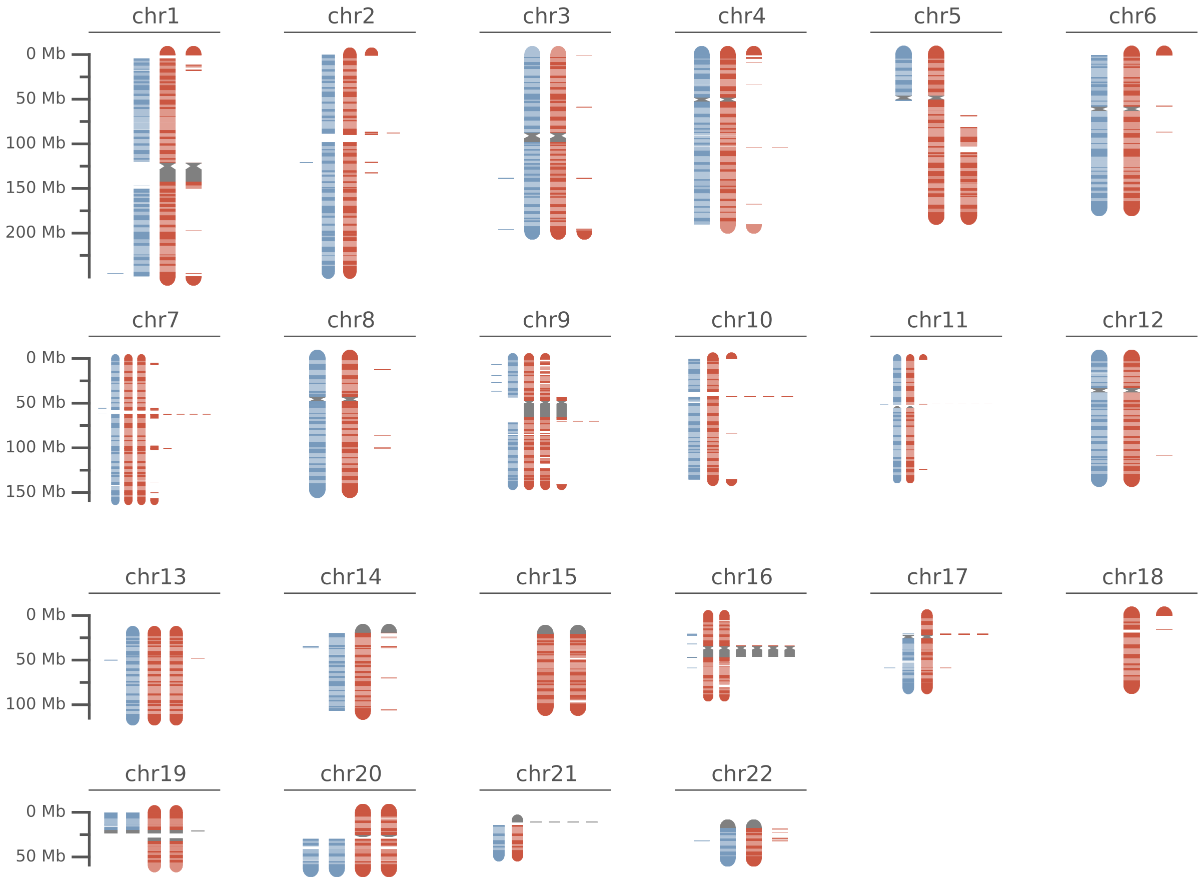

Site of Origin Prediction

About this analysis: Using RNA expression data, this analysis predicts a primary site of origin for the patient's tumor using machine learning. In addition, the most similar samples (by Spearman correlation) to the patient's tumor are identified from a reference cohort of over 8,000 tumor samples. *See Methods section for more details.*

The predicted site of origin for this patient's tumor is **Colorectal** with a False Discovery Rate (FDR) < 8.93e-03 (i.e. a false positive result did not occur in 112 cases). A figure describing the strength of this prediction's score in a cohort of validation samples is available in the *Appendix*.

t-SNE visualization placing the patient's tumor within the context of 30 tumor types and its 10 most similar tumors

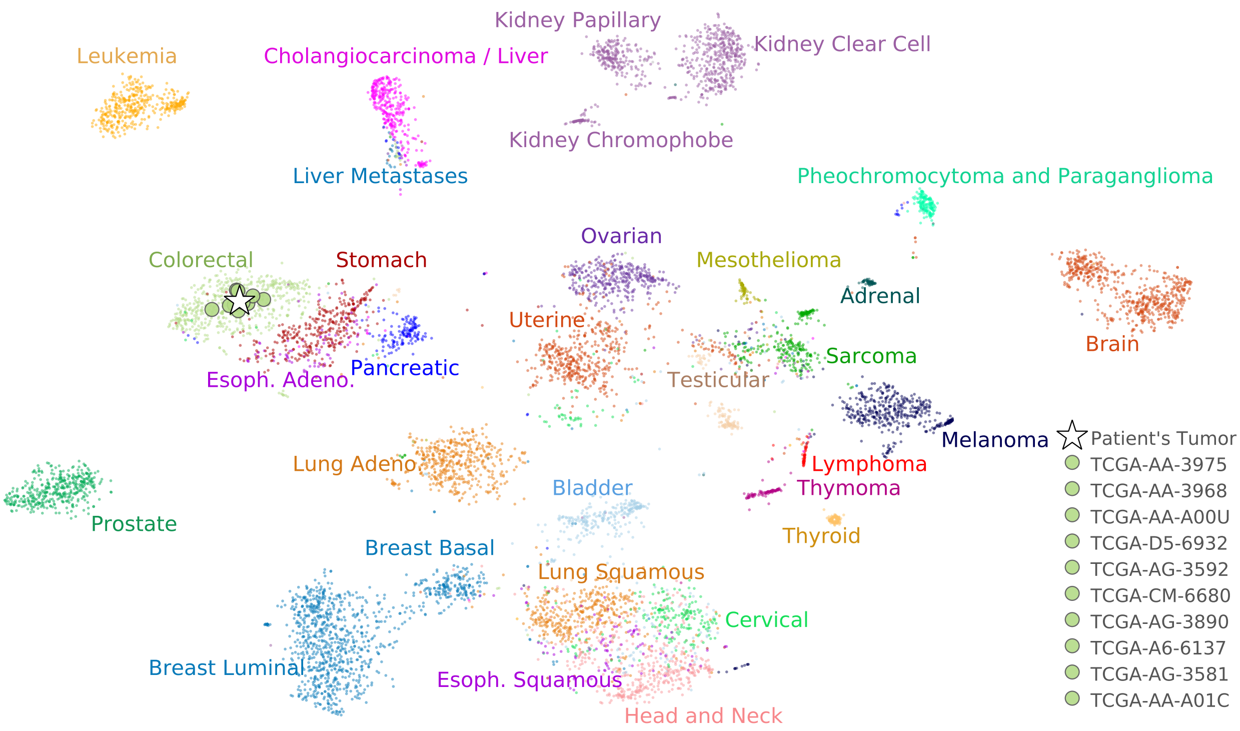

The table below lists the top 10 most similar tumors to the patient's tumor found in the reference cohort. Any additional information for the reference tumor (e.g. Stage, Age at diagnosis) is presented in the *Details* column. These 10 most similar tumors are used to determine the placement of the patient's tumor in the t-SNE figure above.

Top 10 most similar tumors in reference cohort

Rank	Tissue/Site	Correlation	Sample ID	Details
1	Colorectal	0.8743	TCGA-AA-3975	Colon Adenocarcinoma, Stage I (T2-N0-M0), 80 y.o.
2	Colorectal	0.8724	TCGA-AA-3968	Colon Adenocarcinoma, Stage I (T2-N0-M0), 55 y.o.
3	Colorectal	0.8720	TCGA-AA-A00U	Colon Adenocarcinoma, Stage IIIB (T3-N1-M0), 50 y.o.
4	Colorectal	0.8717	TCGA-D5-6932	Colon Adenocarcinoma, Stage IIA (T3-N0-M0), 69 y.o.
5	Colorectal	0.8700	TCGA-AG-3592	Rectum Adenocarcinoma, Stage IIA (T3-N0-M0), 68 y.o.
6	Colorectal	0.8691	TCGA-CM-6680	Colon Adenocarcinoma, Stage IIIB (T3-N2a-M0), 78 y.o.
7	Colorectal	0.8684	TCGA-AG-3890	Rectum Adenocarcinoma, Stage I (T2-N0-M0), 62 y.o.
8	Colorectal	0.8675	TCGA-A6-6137	Colon Adenocarcinoma, Stage IIIB (T3-N1c-M0), 55 y.o.
9	Colorectal	0.8672	TCGA-AG-3581	Rectum Adenocarcinoma, Stage I (T2-N0-M0), 63 y.o.
10	Colorectal	0.8668	TCGA-AA-A01C	Colon Adenocarcinoma, Stage IIIA (T2-N1-M0), 75 y.o.

Inferred Hormone Receptor Status

About this analysis: Gene expression levels derived from RNA sequencing data is used to infer hormone receptor status for the estrogen (ER), progesterone (PR), and Her2 receptors. Thresholds for receptor positivity were determined by comparing gene expression levels to IHC-based receptor calls in a large set of TCGA breast cancers. *See Methods section for more details.*

Inferred receptor status is based on previously observed RNA sequencing and IHC results. The RNA sequencing data from this patient is consistent with the receptor status **ER- / PR- / Her2-**. Additional information is provided in the table below.

Inferred Receptor Status

Receptor	Inferred Status	Expression State	TPM	Threshold
Estrogen receptor	**ER-**	Low/Normal	0.47	17.60
Progesterone receptor	**PR-**	Low/Normal	0.20	2.00
Her2 receptor	**Her2-**	Low/Normal	80.12	240.50

Breast Cancer Subtyping

About this analysis: Gene expression levels derived from RNA sequencing data are used to predict to which of the 5 major breast cancer subtypes (Basal, Luminal A, Luminal B, Her2+, and Normal-like) it is most similar by a model trained on a large set of breast cancer datasets and their corresponding PAM50 subtype calls. *See Methods section for more details.*

The patient's breast cancer subtype was predicted to be **Her2+**. This overall subtype prediction and the probabilities of the sample being associated with each of the 5 major breast cancer subtypes are provided in the table below.

Intrinsic breast cancer subtype prediction

Subtype Prediction	Basal	Luminal A	Luminal B	Her2+	Normal-like
**Her2+**	0.0	0.0	0.0	100.0	0.0

Note: The reported probabilities will always sum to 100 even in settings where prediction of breast cancer subtype is inappropriate, such as applying this model to non-breast cancers.

Colorectal Consensus Molecular Subtype (CMStype)

This patient's CRC CMS subtype is predicted to be **CMS2** (Canonical). This subtype is associated with epithelial, chromosomally unstable, marked WNT and MYC signaling activation, and is found in ∼37% of the CRC population.

CMS Subtype Prediction Probabilities

Subtype Prediction	CMS1	CMS2	CMS3	CMS4
CMS2	0.11%	63.81%	0.04%	36.04%

Expression analysis

A total of 26,465 genes have TPMs estimates according to the methods described below. The table below lists up to 10 cancer genes with the highest gene-level TPM estimates.

Top 10 cancer genes with highest TPM estimates.

Rank	Gene	Status	TPM
180	*RPL10*	Over-expressed	1,504.73
191	*RPL5*	Normal	1,333.59
233	*NPM1*	Over-expressed	933.30
253	*GNAS*	Normal	788.55
258	*ATP1A1*	Normal	753.28
286	*CALR*	Normal	620.92
371	*JUN*	Normal	468.10
388	*AXIN2*	Over-expressed	443.83
395	*RNF43*	Over-expressed	433.60
433	*HIST1H3B*	Over-expressed	388.28

Immunotherapy Markers

About this analysis: Mutational burden, microsatellite instability, mutation signatures, and gene expression of immune checkpoint-related genes are reported here to assess the viability of an immunotherapy approach. *See Methods section for more details.*

Several different metrics correlated to immunotherapy sensitivity are reported below. Further details about specific genes related to immunotherapy sensitivity are described in the methods. The analysis discovered 3 indicators that suggest sensitivity to immunotherapy, summarized in the table below.

Immunotherapy sensitivity markers

Marker	Status	Immunotherapy Permissive
APOBEC/POLE Mutation Signatures	0 of 3 significant POLE/APOBEC signatures found.	No
MSI Status	High degree of microsatellite instability (MSI+).	Yes
Non-Synonymous Mutation Count	233 non-synonymous mutations (11.0 mutations per Mb)	Yes
Immune Checkpoint Expression	3 of 9 immune checkpoint genes are over-expressed.	Yes

Expression of immunotherapy-related genes

Gene	Protein	Immune Function	Expression Status	TPM	Cutoff (TPM)	Median (Clinical, TPM)
IDO1	IDO	Escape signaling	**High**	178.02	62.49	109.56
HAVCR2	TIM-3	Checkpoint	-	42.34	42.63	36.45
C10orf54	B7-H5	Checkpoint	-	19.46	68.74	27.28
CTLA4	CTLA-4	Checkpoint	**High**	7.73	3.89	2.94
PDCD1	PD-1	Checkpoint	**High**	6.88	4.22	2.98
LAG3	LAG-3	Checkpoint	-	5.55	10.09	4.68
CD274	PD-L1	Checkpoint ligand	-	4.52	13.01	3.88
PDCD1LG2	PD-L2	Checkpoint ligand	-	2.55	10.51	4.99
VTCN1	B7-H4	Checkpoint	-	0.08	174.74	1.53

Note: TPM cutoffs are set according to each gene's 95th percentile expression level measured within a cohort of adjacent-normal issues sequenced by TCGA.

Immune Cell Status

About this analysis: The average expression levels of 23 immune cell signatures are computed using RNA sequencing data. The inferred state of each signature is derived using a background distribution of samples within similar ICD10 categories (when an ICD10 code for the sample is available). Elevated expression of immune cells in the tumor biopsy may suggest an immunotherapy approach could be effective. *See Methods section for more details.*

Since this patient's tumor does not have an assignable ICD10 code, the inferred immune cell infiltration data presented in the table below is based on a background distribution of all observed unassignable samples.

Inferred immune cell infiltration status

Cell Type	Inferred Status	Mean Exp.	Background Dist.	Overall Z-score
Dendritic Cells (DC)	Normal	1.74	2.04 +/- 0.77	-0.40
Type 1 T Helper Cells (Th1)	Normal	1.21	1.21 +/- 0.59	-0.01
Natural Killer Cells (NK)	Normal	2.06	1.92 +/- 0.38	0.36
Helper T Cells	Normal	2.95	3.00 +/- 0.64	-0.09
B Cells	Normal	1.45	1.11 +/- 1.14	0.30
Macrophages	Normal	2.67	3.32 +/- 0.91	-0.72
T Cells	Normal	3.10	2.55 +/- 1.25	0.44
Mast Cells	Normal	1.38	1.59 +/- 0.75	-0.28
Immature Dendritic Cells (iDC)	Normal	1.01	1.52 +/- 0.47	-1.09
CD8 T Cells	Normal	1.93	1.97 +/- 0.92	-0.05
Eosinophils	Normal	2.27	2.06 +/- 0.46	0.45
NK CD56bright Cells	Normal	1.52	1.81 +/- 0.64	-0.45
NK CD56dim Cells	Normal	2.91	2.84 +/- 0.84	0.09
Regulatory T Cells (Treg)	Normal	2.41	1.60 +/- 0.79	1.02
Type 2 T Helper Cells (Th2)	Normal	2.05	2.20 +/- 0.47	-0.32
T Helper 17 Cells (Th17)	Normal	2.36	1.81 +/- 0.59	0.94
Neutrophils	Normal	1.95	2.00 +/- 1.15	-0.05
T Effector Memory Cells (Tem)	Normal	2.77	3.35 +/- 0.53	-1.09
T Follicular Helper Cells (TFH)	Normal	3.05	3.14 +/- 0.87	-0.11
T Central Memory Cells (Tcm)	Normal	2.89	3.14 +/- 0.72	-0.34
Activated Dendritic Cells (aDC)	Normal	2.82	2.81 +/- 0.60	0.02
Gamma Delta T Cells (Tgd)	Normal	1.75	2.28 +/- 0.68	-0.78
Plasmacytoid Dendritic Cells (pDC)	Normal	2.73	2.59 +/- 0.83	0.17

Detection of Viral Sequence

No viral sequences were detected in the patient's sequencing data.

Complex Phenomena

The following complex phenomenon was detected: *kataegis*. The figure below indicates in red the locations in the genome where these phenomena were detected.

Structural variants, copy number, and mutational distance across the tumor genome. Locations of complex phenomena are displayed in red. Due to large numbers of SNVs in this tumor genome, the mutational distance plot displays a small random sample of all mutations in the tumor.

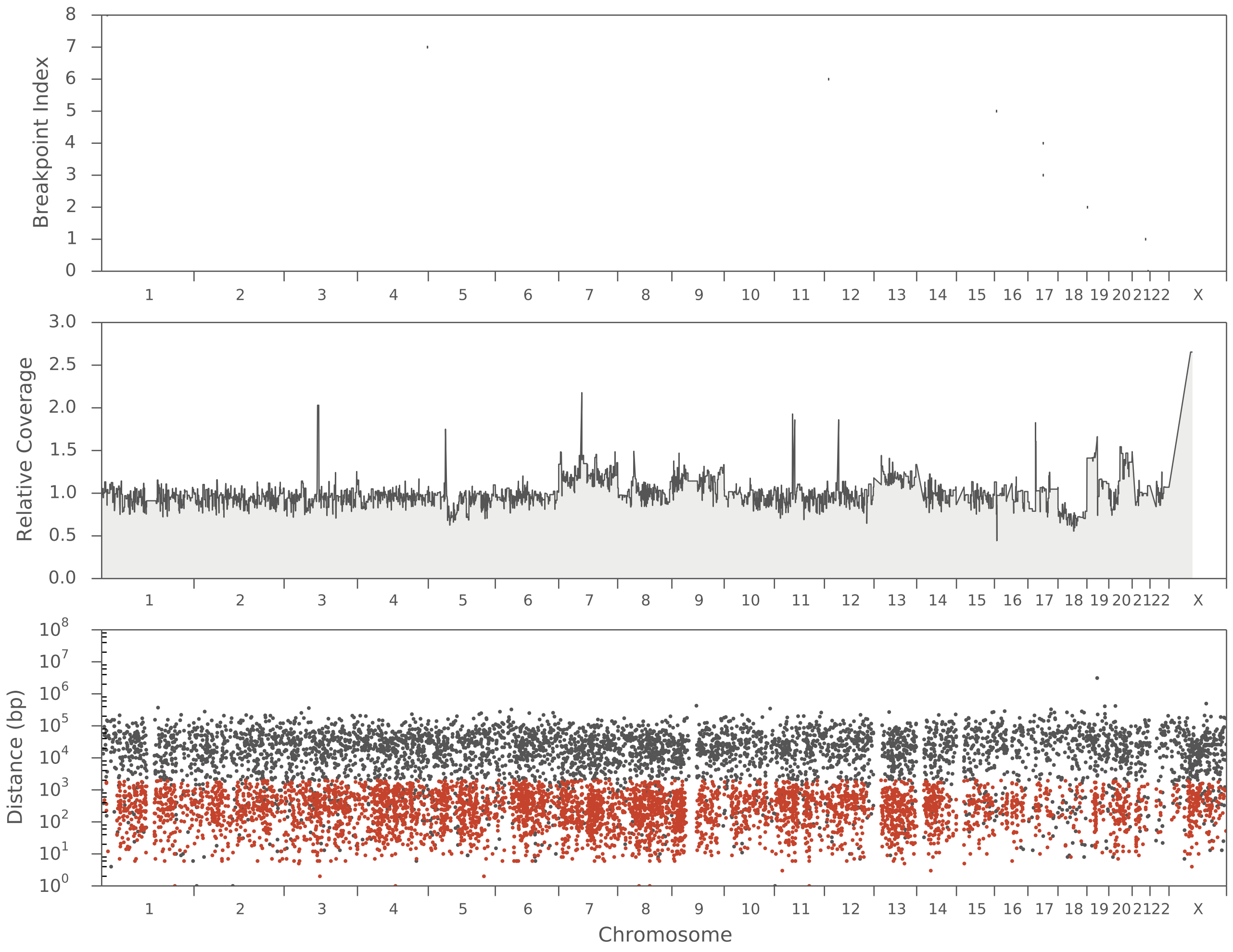

Mutational Signatures

About this analysis: Using non-negative matrix factorization (NMF) on counts of mutated triplets detected in the tumor sample, the contributions of 30 mutational signatures characterized by the Sanger Institute are estimated. These mutational signatures can identify defects in DNA left by exposure to carcinogens (e.g. tobacco smoke), disfunction of DNA repair mechanisms (e.g. BRCA1/2 or MMR genes), and/or the activity of a variety of mutational processes (e.g. APOBEC / AID). *See Methods section for more details.*

In this sample, a total of 553,042 single nucleotide variants were identified. A figure plotting the distribution of mutations according to their genomic contexts, can be found in the appendix. The table below shows the 6 mutational signatures believed to be active in this patient.

Active mutational signatures identified in this sample.

Signature	# SNVs	% of Total	Caused by	Associated with	Common tumor types
Signature 12	149,408	27.0	Unknown	Transcriptional strand bias for T>C mutations	Liver
Signature 16	119,550	22.0	Unknown	Strong transcriptional strand bias for T>C mutations	Liver
Signature 3	87,763	16.0	BRCA1/BRCA2 mutations	Failure of double-strand break-repair by homologous recombination	Breast, Ovarian, Pancreatic
Signature 1	77,261	14.0	Spontaneous deamination of 5-methylcytosine	Age of cancer diagnosis	All
Signature 9	56,305	10.0	DNA repair by polymerase eta	Activity of AID during somatic hypermutations	CLL, B-Cell Lymphoma
Signature 11	17,238	3.0	Exposure to alkylating agents	Transcriptional strand bias for C>T mutations	Melanoma, Glioblastoma

The plot below describes the contribution of 3 of the top ranked mutation signatures versus mutation allele fraction. With the assumption that mutations with higher allele fractions are likely present in a larger proportion of the tumor population (and thus likely to have occured earlier in the tumor's development), this plot may show how the activity of these mutational signatures changed over time.

Top Ranked Mutation Signatures vs. Allele Fraction

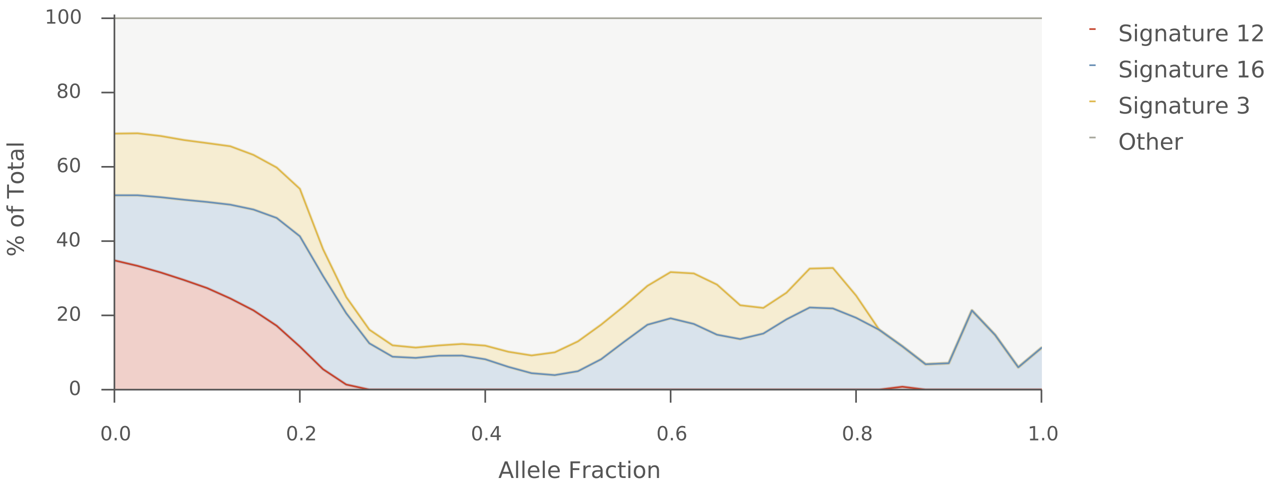

Activity of APOBEC/AID Family

About this analysis: In tumors with large contributions of the APOBEC/AID mutational signatures, this analysis attempts to find genetic factors that may explain the predicted activity, such as variants in the *APOBEC3* or *AID* family of genes, deletion of *APOBEC3B*, SNPs associated with its activity, and the presence of viruses. *See Methods section for more details.*

Fewer than 5% of somatic mutations in the tumor are attributed to APOBEC/AID-related signatures, so it is unlikely that APOBEC/AID gene family is active in this tumor.

The patient's sequencing data were analyzed for multiple genetic factors known to be associated with increased activity of the APOBEC/AID family of cytidine deaminases. The results of the analysis are provided in the table below.

Genetic factors associated with APOBEC/AID mutational signatures

Feature	Value	Interpretation
% of Mutations	1.0%	APOBEC/AID family not active in tumor
APOBEC3B Relative Coverage	1.22	No evidence of *APOBEC3B* deletion
Somatic Variants	None found	No evidence of somatic disruption to *APOBEC3/AICDA* genes
Germline Variants	None found	No evidence of germline disruption to *APOBEC3/AICDA* genes
Associated SNP	rs1014971 (100% AF DNA+)	SNP is associated with APOBEC/AID activity
Viral Detection	None found	No evidence of assayed viral sequences

RNA Fusion Analysis

About this analysis: Transcriptome-aligned RNA sequencing data is used to identify potential fusions between one of 74 genes commonly found in oncogenic fusions and any other gene in the human transcriptome, In addition, the intragenic fusions (splice variants) EGFRvIII and MET exon 14 skipping are identified by this method.Only *functional* fusions are reported here, herein defined as fusion transcripts with open reading frames extending from the reference start codon of the fusion's upstream partner to the reference stop codon of the downstream partner. Fusions must have greater than 8 reads supporting the fusion junction. Lastly, fusions where the upstream gene contribution consists solely of sequence from the untranslated region (UTR) must have some level of support in the DNA to be reported here. *See Methods section for more details.*

The analysis identified 3 fusion transcripts comprised of at least one of 74 genes commonly observed in oncogenic fusions in the RNA sequencing data. A summary of these results, in addition to a count of spanning read support for each fusion transcript present in the DNA sequencing data, is provided in the table below.

Summary of fusions identified in RNA sequencing data

Fusion	Protein Description	Exon Composition	RNA Support	DNA Support
TCF3 Internal	(19;19)(*TCF3*:p.M1_G49; *TCF3*:p.G100_*655)	*TCF3*(e1-3) + *TCF3*(e6-19)	53	T:221, N:53 *
TMPRSS2 Internal	(21;21)(*TMPRSS2*:p.M1_M109; *TMPRSS2*:p.V149_*493)	*TMPRSS2*(e1-4) + *TMPRSS2*(e6-14)	17	T:350, N:65 *
ETV4 Internal	(17;17)(*ETV4*:p.0?; *ETV4*:p.?) *	*ETV4*(e1-1) + *ETV4*(e3-13)	16	T:295, N:78 *

* Fusion is composed of UTR sequence from the upstream gene and a new translation initiation site was predicted to produce a functional fusion gene. Without an ability to confirm the actual protein composition, the predicted fusion is described as p.0? (probably no protein) and p.? (unknown protein) for the upstream and downstream gene contributions, respectively.
* The spanning read supports are provided in the format "T:X, N:Y" where T and N refer to the tumor and matched-normal DNA sequencing datasets, respectively.

Junction Analysis

About this analysis: Read support for junction sequences specific to 3 clinically important gene fusions and alternatively spliced genes is quantified from RNA sequencing data. Junctions supported by the sequencing data are reported here along with their potential therapeutic implications. *See Methods section for more details.*

No support for the 3 junction sequences analyzed here were discovered in the patient's sequencing data.

Structural variants

Out of the 13 structural variants identified that overlap at least one gene, no structural variants were found to affect any cancer genes. The full list of structural variants can be found online.

The circular genome plot for this sample can be found below, which plots copy number, allele fraction, and structural variants across the entire tumor genome

Provenance

About this analysis: Genotypes at dbSNP loci are analyzed between samples believed to be from the same person (e.g. tumor and normal). A total of 1,000 such sites are compared between samples, and the number of *incompatible* (mismatched) genotypes that exist between samples are tallied. If the percentage of incompatible genotypes exceeds 3% for Tumor vs. Normal DNA (or 5% for RNA vs. Normal DNA when RNA is available), the samples likely belong to different people. *See Methods section for more details.*

The estimated sample similarities presented in the table below indicates that all samples belong to the same patient.

Provenance of patient's tumor DNA, matched-normal DNA, and tumor RNA samples

Comparison	# dbSNP	# Identical	# Compatible	# Mismatched	% Similarity
Tumor vs. Normal	1,000	934	61	5	99.50
RNA vs. Normal	1,000	806	184	10	99.00

Homozygosity of X

About this analysis: The percentage of germline dbSNP loci on chromosome X are calculated to estimate its homozygosity. It is expected that females have two copies of chromosome X and are thus heterozygous for a significant fraction of alleles, while males have one only one copy of chromosome X and are thus homozygous. Ensuring the known sex of the patient is consistent with the observed homozygosity of chromosome X is another method, orthogonal to *Provenance*, that helps increase confidence that a sample mixup has not occurred. *See Methods section for more details.*

Out of 65,370 dbSNP loci analyzed, 64,694 sites were homozygous in the tumor and 63,845 sites were homozygous in the matched-normal. Both tumor and matched-normal samples exhibit similar chromosome X homozygosity.

Summary of homozygosity estimates for patient's samples

Sample	# dbSNP	# Homozygous	% Homozygous
Tumor	65,370	64,694	99.0
Normal	65,370	63,845	97.7

Contrast Summary

Summary of variants detected in sample

Variant Type	Count
Intergenic	396,599
Intron	191,033
NonCoding	31,951
Missense	211
Silent	131
Nonsense	9
Frame Shift Deletion	4
In-Frame Insertion	3
In-Frame Deletion	3
Frame Shift Insertion	2
SpliceSiteCDS	1

Distribution of detected variants

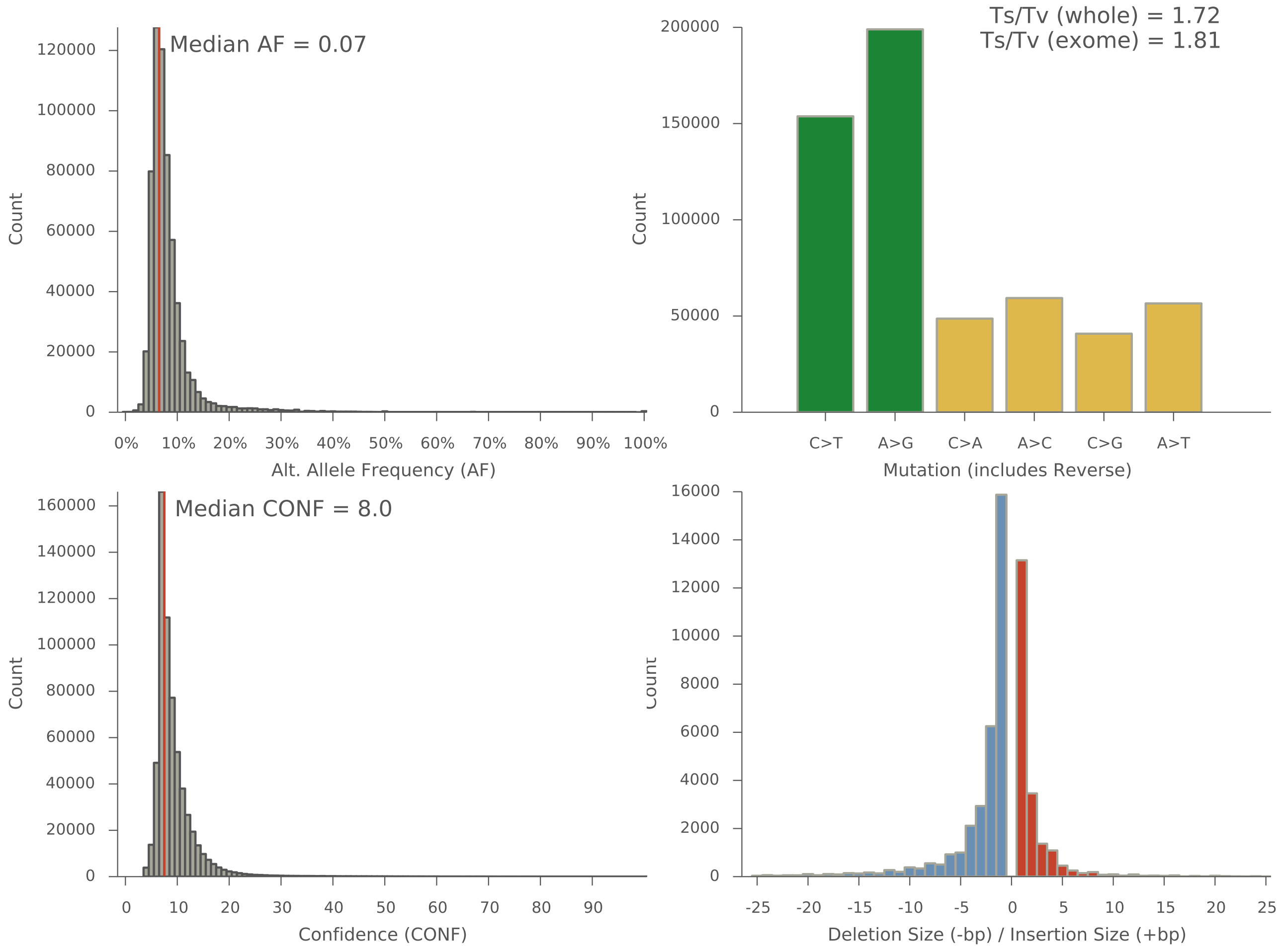

Sequence Information

About this analysis: Numerous quality metrics are collected on the raw sequencing data generated for the patient samples. These metrics include: average coverage in the whole genome & exome, % mapped & duplicate reads, bias in GC-rich regions, and base composition and quality vs. position in read. In addition, the minimum coverage depths for 75, 90, and 99% of bases in coding regions of known cancer genes are determined. *See Methods section for more details.*

Summary of sequencing data

Sample	Analyte	# Reads	% Mapped	% Duplicates	Genome / Exome Cov.	75 / 90 / 99% Bases Cov.
Tumor	WGS	1,639,683,633	99.60	12.60	62x / 73x	32x / 24x / 16x
Normal	WGS	732,523,118	99.60	5.00	32x / 31x	27x / 22x / 11x
Tumor	RNA	514,240,994	65.10	0.00	3x / 143x	43x / 6x / 0x

Known cancer genes with lowest percentage of coding bases covered by > 10 reads in tumor sample

Gene	Length (bp)	# Covered	% Covered
DAXX	2,223	2,150	96.7
MAGI2	4,368	4,250	97.3
BTK	1,980	1,928	97.4
MAP3K1	4,539	4,459	98.2
ATRX	7,479	7,432	99.4
PBRM1	4,749	4,730	99.6
KDM6A	4,362	4,351	99.7
KMT2A	11,919	11,915	100.0
ABL1	3,450	3,450	100.0
ABL2	3,549	3,549	100.0

Sample Contamination

About this analysis: The fraction of somatic variants occurring at common sites of human polymorphism is used to detect if the tumor sample is contaminated with the DNA of another individual. Furthermore, the number of somatic variants detected in the matched-normal is used to determine if the matched-normal sample was contaminated with DNA from the tumor sample. *See Methods section for more details.*

Out of 619,947 somatic variants analyzed, 6,018 variants occurred at common dbSNP markers and 801 variants were present in the matched-normal. The low percentage of common sites (1.0%) indicates that the tumor sample is not contaminated with DNA from an unrelated individual. The low percentage of somatic sites (0.1%) present in the matched-normal indicates that it is not contaminated with DNA from the tumor sample.

Summary of sample contamination metrics

# Somatic	# Common	% Common	Common AF	# RNA	# in Normal	% in Normal	In Normal AF
619,947	6,018	1.0	0.08	2	801	0.1	0.12

Cumulative Percentage of Common Sites vs. Allele Fraction. 'Minimum Acceptable Allele Fraction = 0.000.

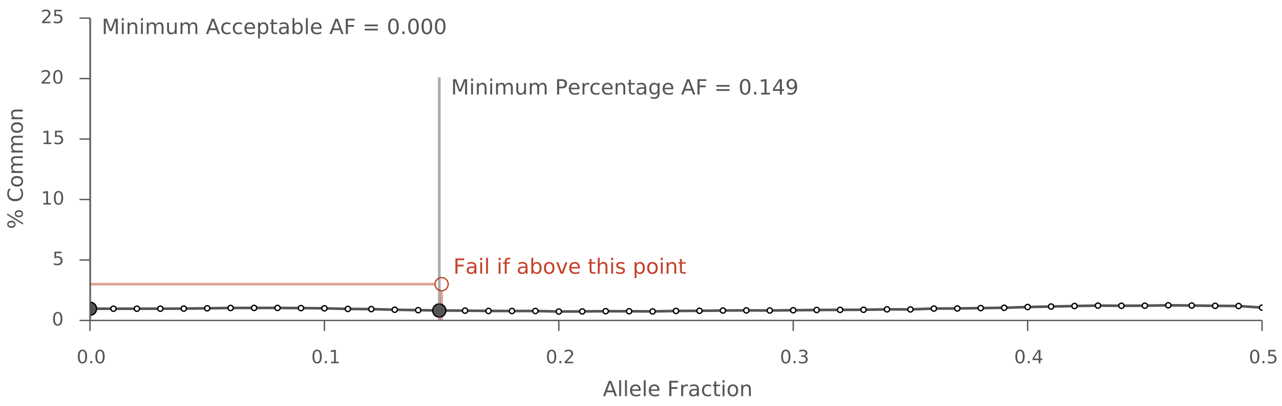

RNA Quality

About this analysis: A set of quality metrics specific to RNA sequencing data is collected and analyzed to determine if the RNA sequencing data is of sufficient quality for the purposes of verifying mutant allele expression. Note that TPM levels are only used to classify gene expression status and does not influence what is presented here. *See Methods section for more details.*

RNA coverage metrics required for this analysis is not available for this patient.

About This Test

DNA sequencing libraries were prepared for tumor and matched-normal samples using the KAPA Hyper prep kit and sequenced on the Illumina sequencing platform.

DNA sequencing data is aligned to Genome Reference Consortium Human Build 37 (GRCh37, aka hg19) using the programs listed in the methods section below. Tumor vs. matched-normal variant analysis was performed using the NantOmics Contraster analysis pipeline to determine somatic & germline single nucleotide variants, insertions & deletions, and identify highly amplified regions of the tumor genome.

Small variants were annotated with base-level PhastCons conservation scores, population allele frequencies from dbSNP (Build 142), and for their predicted impact to genes. Each small variant predicted to alter the protein sequence of a gene is further analyzed by a proprietary *de novo* assembly algorithm that realigns all reads surrounding the variant from both tumor and matched-normal samples to increase confidence that the detected somatic or germline variant is real.

RNA-Seq libraries were prepared for the tumor sample using KAPA Stranded RNA-Seq with RiboErase kit and sequenced on the Illumina sequencing platform. RNA sequencing reads were aligned by `bowtie2` using default parameters to the RefSeq transcriptome and analyzed by `RSEM`. Transcriptome-based alignments are converted to reference genome coordinates to detect expression of variants identified in the DNA sequencing data.

Normalized gene-level TPMs are used to determine if the gene is High or Over-Expressed based on cutoffs derived from orthogonal technologies and/or datasets. Four techniques for establishing cutoffs were used, including optimizing TPM concordance for preset levels, identifying cutoffs that maximize concordance between platforms, selecting cutoffs that maximize positive predictive value (PPV), or utilization of alternative datasets with outcomes or phenotypes of interest. Orthogonal testing techniques included proteomics selected reaction monitoring (SRM) technology and alternative RNA-Seq library preparations (poly-A).

Note that the minimum tumor purities listed in the table below refer to purities measured post-microdissection. Tumor specimens with lower purities are often acceptable provided that microdissection can enhance the purity to exceed the purities specified below.

Summary of performance characteristics

Validated to Report	Performance	Minimum Acceptable Criteria
Somatic, Clonal SNVs (+ RNA Expression)	>95% Sensitivity, >99% Specificity	30% Tumor Purity
Somatic, Clonal Insertions & Deletions (+ RNA Expression)	>95% Sensitivity, >99% Specificity	30% Tumor Purity
Germline SNPs (+ RNA Expression)	>95% Sensitivity, >99% Specificity	N/A
Germline Insertions & Deletions (+ RNA Expression)	>95% Sensitivity, >99% Specificity	N/A
Amplifications	>95% Sensitivity, >99% Specificity	30% Tumor Purity
Fusion Genes	100% Sensitivity, 100% Specificity	20 TPM
Microsatellite Instabilty (MSI)	100% Sensitivity, 100% Specificity	13% Tumor Purity

Curated Findings

Version 2.3

Findings are curated from multiple sources, such as primary literature and FDA drug labels. When genomic and/or transcriptomic biomarkers associated with these findings are detected in the sequencing data of the patient (somatic or germline), the finding is summarized by this analysis.

Molecular Oncology Findings

Version 2.1

Variants occuring in 239 known cancer genes and those deemed treatable in other analyses of this report are reported here. Known cancer genes are classified as tumor suppressors or oncogenes using data available from COSMIC Cancer Gene Census [1].

Only small variants predicted to be Pathogenic or Likely Pathogenic are presented here, using the heuristic procedure described in *Additional Mutational Analysis*. All variants occuring in treatable genes will be reported.

Mutational burden is classified as HIGH in tumors with 200 or more non-synonymous mutations, which is associated with clinical benefit of anti-PD-1 therapy [2]. Tumors with fewer than 200 non-synonymous mutations are classified with LOW mutational burden.

Protein Analysis

Version 2.2

22 clinical protein markers in the following genes are measured using mass spectrometry: Her2, ALK, ROS1, PDL1, EGFR, AR, hENT1, TOPO1, TOPO2A, FRalpha, SPARC, TUBB3, ERCC1, MGMT, RRM1, Her3, MET, AXL, MSLN, FGFR2, IGF1R, RON. An additional 4 protein markers are measured by the LungAdenoPlex assay for lung cancers: p63, K7, K5, TTF1. When available, p16 protein expression is measured to indicate potential HPV infection in head & neck, cervical, anal, and rectal cancers, and KRAS protein expression is measured to predict poor prognosis in gastroesophageal and endometrial cancers.

Genes Associated with Chemotherapy Response

Version 1.3

Expression levels for the following 8 genes associated with differential response to chemotherapy are reported here: TOP1 (TOPO1), TYMP, SLC29A1 (hENT1), FOLR1 (FR-alpha), TOP2A (TOPO2A), RRM1, TUBB3, MGMT. For each gene, the empirically-derived cutoff and median expression level within previously-assayed clinical samples are provided. Expression status is classified as **High** if the sample's TPM is higher than its associated cutoff. The TPM cutoffs were optimized to best approximate the following proteomics-based cutoffs: TOPO1 = 2,075, TYMP = 2,600, hENT1 = 338, FR-alpha = 1,300, TOPO2A = 1,570, RRM1 = 390, TUBB3 = 1,000, and MGMT = 200 amol/μg. This approximation to proteomics is presented so that comparison of previously-tested samples may be performed with the caveat that new samples will have inherent heterogeneity.

Additional Mutation Analysis

Version 2.1

All somatic variants are classified into the following 5 categories: "Pathogenic", "Likely Pathogenic", "Variant of Unknown Significance", "Likely Benign", and "Benign". The variant's category is determined using a combination of variant class (e.g. Missense), amino acid change, PhastCons conservation score of the mutated site, gene type (i.e. Oncogene, Tumor Suppressor, or neither), driver status (e.g. Driver Gene), variant allele frequency in the population from dbSNP, and if the variant is located inside of a gene's mutational hotspot.

The disruption of a particular amino acid change is calculated according to a Conservation-controlled Amino acid Substitution Matrix (CASM) score [11], with parameters estimated using Five3 variant calls on >5,000 TCGA tumor exomes and their matched-normals. PhastCons was downloaded from the UCSC Genome Browser. Gene type is obtained using data from the COSMIC Cancer Gene Census [1]. Driver status is obtained from a pan-cancer publication across 15+ TCGA cancer types [12]. Clusters of mutations were discovered using OncodriveCLUST on the variant calls made on >5,000 TCGA tumor exomes [13]. Variants found in the COSMIC database (release v76) are annotated with the number of COSMIC samples harboring mutations that cause the same protein change [1].

Mutation clonality is determined using the purity and ploidy estimates produced by *Cytogenetic Analysis*, when available. These estimates are used to transform the local relative coverage of the copy number segment harboring the mutation into somatic copy number, which is used to determine the posterior probability that at least 75% of tumor cells harbor mutation [14]. A mutation is deemed *clonal* if its posterior probability exceeds 0.75, *subclonal* if less than 0.25, and otherwise undetermined.

Design of the figure comparing this tumor sample's exonic mutation rate to mutation rates of tumors sequenced by TCGA is attributed to Gad Getz and his colleagues at the Broad Institute.

RNA Rescue: COSMIC SNVs in Cancer Genes

Version 1.0

Raw genome-aligned RNA sequencing data is scanned for support of single nucleotide variants in the COSMIC database (build v55) in known oncogene and tumor suppressors that lack support in the patient's DNA sequencing data. To reduce false positives from such an approach, the support in the RNA sequencing data must be significant, with at least 4 unique reads, representing 25.00% of reads.

Secondary Screening for Cancer Predisposition

Version 1.6

DNA sequencing data from both tumor and normal tissues are scanned for relevant germline variants (i.e. nonsense SNVs and frame-shifting insertions & deletions) in the following 22 genes implicated in increased risk of developing cancer: APC, BMPR1A, BRCA1, BRCA2, MEN1, MLH1, MSH2, MSH6, NF2, PMS2, PTEN, RB1, SDHB, SDHC, SDHD, SMAD4, STK11, TP53, TSC1, TSC2, VHL, WT1, which are included in ACMG's recommendations for reporting incidental findings [15]. Variants must be sequenced to a minimum depth of 10 reads and have a minimum alternate allele fraction of 0.25 in the normal sequencing data to be reported.

Known pathogenic variants found in BRCA1/2 are identified using a database of clinically important BRCA1/2 germline variants extracted from NHGRI's Breast Cancer Information Core (BIC) [16].

Microsatellite Instability

Version 1.2

Instability of microsatellite repeats is estimated using the method described here [17]. A set of 2,848 microsatellites consisting of homopolymer repeats were analyzed for a statistically significant increase in the number of length polymorphisms in both tumor and matched-normal (if available) sequenced. The background mean (μ) and standard deviation (σ) of the number of length polymorphisms for each microsatellite locus were computed across approximately 5,000 blood and solid normal exomes sequenced by TCGA comprising 18 different cancer types. Loci covered by fewer than 30 reads are excluded from the analysis. For each microsatellite locus, the number of differently-sized repeats are counted for each sample. Repeats with read support exceeding 5% of the read support of the maximally-supported repeat are tallied for a total count of differently-sized repeats, n. The total number of unstable microsatellites is counted in each sample, where a given microsatellite i is deemed unstable if $n_i > \mu_i + 3\sigma_i$. The percentage of unstable loci is calculated for the tumor and matched-normal. The differential is then determined by subtracting the percentage of unstable loci in the normal sample from the percentage of unstable loci calculated in the tumor. A tumor is considered to demonstrate microsatellite instability (MSI) when the differential exceeds the threshold specified in the results.

Disruptive alterations to DNA repair genes (MLH1, MSH2, MSH6, PMS2) are presented in this analyis. Alterations are restricted to germline nonsense and frame-shifting insertions or deletions, somatic variants classified as *Pathogenic* or *Likely Pathogenic* (see *Mutation Analysis* for details), and somatic gene losses.

Copy number analysis

Version 1.5

Relative coverage and majority allele fraction of the tumor sample versus its matched normal were estimated using a single-pass segmentation algorithm that merges fixed-width contiguous regions of the genome unless the estimates of the relative coverage and majority allele fraction (when available) of the regions differ in a statistically significant manner (i.e. greater than 3 standard deviations). The regions outputted by the single-pass segmentation algorithm are corrected for estimated GC bias. Variable regions with the weakest support are merged with the neighboring region that best matches the region's estimates, and then the newly neighboring regions are merged using the same significance criteria as before. This last step is iteratively performed until regions can no longer be merged together.

Copy number status for a given region is defined as "Amplification" if $\log_2(rc) > 1.0$, "Moderate Amp." if $\log_2(rc) > 0.25$, "Loss" if $\log_2(rc) < -0.25$, and "Normal" otherwise, where rc is the region's estimate of relative coverage normalized by the total read counts of tumor and matched-normal. Weakly supported segments (WGS: $rc_n < 200$ or $af_n < 100$, Exome: $rc_n < 20$ or $af_n < 10$) are not plotted unless the segment harbors at least one cancer gene and is among the top 10 segments with highest or lowest relative coverage.

Copy number is computed from relative coverage estimates using the purity and ploidy estimates produced by *Cytogenetic Analysis*, when available.

Oncogenes that experience gains in copy number ("Moderate Amp." or "Amplification") and tumor suppressor genes that experience losses are highlighted as significant findings if the genes are deemed as "High Confidence Drivers" by a study of the mutations detected in 3,000+ exomes sequenced by TCGA [12].

Pharmacogenomics Screening

Version 1.1

DNA sequencing data from both tumor and normal tissues are scanned for relevant germline variants (i.e. nonsense SNVs and frame-shifting insertions & deletions) in the following 22 genes implicated in increased toxicity of anti-cancer drugs: *CEP72, CYP2C8, CYP2D6, CYP3A5, DPYD, F5, FCAMR, G6PD, HLA-DQA1, HLA-DRB1, ITPA, MTHFR, NUDT15, RARG, SLC28A3, SLCO1B1, SV2C, TLR4, TPMT, UGT1A1, UGT1A6, UGT1A9*. Variants must be sequenced to a minimum depth of 10 reads and have a minimum alternate allele fraction of 0.25 in the normal sequencing data to be reported.

Status of HRD-Related Genes

Version 1.0

A set of genetic factors attributed to homologous recombination deficiency (HRD) are scanned for in the patient's sequencing data. These factors include: germline & somatic nonsense and frame-shifting variants, copy number alterations, and expression levels in genes related to HRD: *BRCA1*, *BRCA2*, *PALB2*, *RAD51C*. BRCA1/2 mutation signature is predicted as active if more than 5% of somatic mutations are attributed to its mutational signature.

Cytogenetic Analysis

Version 1.12

This analysis uses the copy number segments described in *Copy Number Analysis* to estimate the amount of normal contamination, α, and tumor ploidy that can be used to transform the relative coverage estimates into allele-specific, integral copy number states where possible.

Best fit parameters for α and tumor ploidy are found by gradient descent. Each round of gradient descent is initialized with random values for α and ploidy and attempts to maximize the joint log-likelihood of the relative coverage, rc, and majority allele fraction, af, estimates weighted according to segment size across the 22 autosomes. Gradient descent is performed in this manner for a minimum of 10 times, and the best fit parameters across all rounds are reported.

In the joint log-likelihood calculation, a set of common allelic states are used to determine the expected relative coverage and majority allele fraction values for each state, given α and tumor ploidy. These states include commonly altered states such as single copy gain $(2,1)$, loss-of-heterozygosity or LOH $(1,0)$, and copy-neutral loss-of-heterozygosity or CN-LOH $(2,0)$, where the numbers in parentheses are the majority and minority allelic copy numbers, (A,B), that describe an allelic state. Additionally, less common states such as balanced amplification $(2,2)$ and subclonal states representing a 50/50 mixture of subclones with and without an altered allelic state are also used.

Tumor ploidy is recalculated using the best fit parameters to transform the original relative coverage estimates into tumor copy number. Ploidy is then calculated as the average of tumor copy number across the whole genome, weighted by the normalized genomic length of each segment.

Site of Origin Prediction

Version 1.0

This report application uses RNA-Seq data to predict the site of origin for a given tumor sample. It uses a reference cohort of RNA-Seq data from clinical and publicly available research samples and compares gene-level transcriptional profiles of this sample and the samples in the reference cohort. Predictions are made based on a subset of most varying genes.

Sample similarity is computed using Spearman correlation of the samples' transcriptional profiles, which is often used in genomic studies to determine nearest neighbors for a given sample. The tumors to which this sample is most similar can help to inform clinical decisions.

Inferred Hormone Receptor Status

Version 1.0

IHC-based receptor calls and corrosponding gene expression values were obtained from TCGA breast cancer datasets [23]. Optimal thresholds in gene expression values for agreement with IHC calls were found using Youden J-index analysis. Using 10-fold cross-validation, the accuracy of this approach was determined to be 94%, 84%, and 85% for ER, PR, and Her2 in held-out TCGA breast cancer samples, respectively. In an external breast cancer cohort, the accuracies were determined to be 83%, 73%, and 86% for ER, PR, and Her2, respectively.

Breast Cancer Subtyping

Version 1.0

PAM50 subtype calls and gene expression levels were obtained from the supplemental information of the TCGA landscape breast cancer paper [23]. This dataset was divided into a 70/30 split of training and testing sets, respectively. The model used here was trained on the training set and achieved >92% subtyping accuracy in the held-out samples of the testing set. The subtype assignments produced by this model are slightly more prognostic than the original 2015 PAM50 labels, and have been validated to be significantly prognostic in two independent breast cancer cohorts.

Colorectal Consensus Molecular Subtype (CMStype)

Version 1.0

The CRC Consensus Molecular Subtypes (CMStypes) were first identified by merging the subtyping efforts upon microarray expression profiles from >4,000 colorectal samples performed by 6 independent research groups. A random forest classifier was then designed to reliably classify unseen samples into these types.

The results shown here are a novel implementation of CMStype classification designed for use with Nant RNAseq data. CMStype labels were obtained for over 1400 clinical cases and used to train a multi-class logistic regression model. This modeling strategy achieves >97% concordance with CMStypes from the original authors in unseen CRC samples.

Expression analysis

Version 1.0

Any available RNA-Seq data for the patient is processed by RSEM [25] to estimate transcripts per million (TPM) and fragments per kilobase of exon per million fragments mapped (FPKM) for each isoform. Gene-level TPM and FPKM estimates are made using a weighted-average of the isoform estimates, weighted by the percentage that RSEM estimates each isoform is expressed among all isoforms in the sample.

Gene-level TPMs are used to determine if the gene is "Over-expressed", "Under-expressed", "Not expressed", or "Normal" using the lower and

upper 5th percentiles of per-gene RSEM TPM values for a collection of RNA-Seq datasets from TCGA normal samples. The expression status for a gene is classified as "Over-expressed" if its TPM exceeds the gene's upper 5th percentile, "Under-expressed" if it less than the lower 5th percentile, "Not expressed" if its TPM value equals zero, or otherwise classified as having a "Normal" expression status. If a gene's upper or lower 5th percentile is unavailable, the expression status for that gene will be classified as "N/A".

Immunotherapy Markers

Version 2.2

Microsatellite instability (MSI) status is determined using the methods described in the the *Microsatellite Instability* section. Total mutation counts reports the number of non-synonymous mutations present within the tumor sample. These metrics has been previously shown to be associated with response to immunotherapy [27].

A patient's RNA-Seq data is examined for expression of immune checkpoints using the methods described in the *About This Test* section. High expression of key immune checkpoint genes, PD-1, PD-L1, PD-L2 and CTLA-4 may indicate active suppression of immune cells by the tumor microenvironment [26]. High expression of IDO1 and TIM-3 may indicate immune tolerance mechanisms employed by the tumor [28, 29]. For each gene, the median expression level within previously-assayed clinical samples is provided.

Mutational signatures are determined by the methods described in the *Mutation Signatures* section. APOBEC- and POLE-related mutation signatures that contribute more than 5% of all mutations observed in the sample are reported here.

Immune Cell Status

Version 1.1

A panel of 109 genes that accurately discriminate between 23 immune cell subpopulations were used as the basis of this analysis [30]. For each of these 23 immune cell signatures, the average expression level of the genes involved in each signature was calculated. Additionally, these mean expression of the 23 immune cell signatures are compared to similar samples (based on ICD10 category, when available) to infer if activation is over or under the expected range for the cancer's tissue type.

Detection of Viral Sequence

Version 1.1

For samples aligned to the sequences of viruses implicated in cancer, the coverage of each viral genome is determined by the median read depth across all positions of the viral genome. Any viral genome with a median coverage greater than 3x in the primary and/or matched-normal samples will be reported, but only those with median coverage greater than 10x will be highlighted.

Complex Phenomena

Version 0.1

Somatic variants identified in the tumor genome are used to detect evidence of three complex phenomena: kataegis, extreme gains in copy number, and clustered rearrangements. Kataegis is a pattern of dense clusters of hypermutated bases, often found near somatic rearrangements. Clustered rearrangements can be evidence of a process called chromothripsis, whereby the genome is shattered into hundreds of pieces that are then randomly put back together, resulting in segments of the tumor genome that are highly-rearranged and with frequent loss of genomic material. When clustered rearrangements are found in a region that also exhibits extreme copy number, this signals the possibility that a double minute chromosome is present in the tumor genome.

Point mutations with scores of at least 10 and separated by no more than 2 Kb from its nearest neighbor are considered candidate mutations in the kataegis analysis. If a region of the genome contains a minimum of 10 candidate mutations and has a density of at least 10 candidate mutations per 100 Kb, it is classified as a potential kataegis event.

Regions exhibiting extreme gains in copy number are defined as those with relative coverage (versus matched normal) exceeding 5.0 over a span of at least 100 Kb.

Structural variants are required to have a minimum support of at least 6 reads with an average mapping quality greater than 30. Clustered rearrangements are defined as a region containing no fewer than 5 structural variants (separated by at least 10 Kb), with a breakpoint density of at least 5 breakpoints per 1 Mb.

Mutational Signatures

Version 1.1

The bases directly adjacent to the mutated site are used to determine the genomic context of the site, which can help to determine if a particular mutagen (e.g. tobacco smoke, exposure to ultraviolet light) or mutational process is active in the sample.

The exposure to each of the 30 signatures identified by Sanger was calculated using non-negative matrix factorization (NMF) on the counts of mutated triplets identified in the tumor sample [33]. "Active" signatures are those that contribute at least 100 mutations (representing a minimum of 2% of all mutations) or greater than than 25% percent of all mutations in the sample.

Activity of APOBEC/AID Family

Version 1.0

A set of genetic factors attributed to increased APOBEC/AID activity are scanned for in the patient's sequencing data. These factors include: germline & somatic nonsense and frame-shifting variants in the related genes, associated SNPs, deletion of *APOBEC3B*, and any detected viruses. The related genes are *APOBEC3A*, *APOBEC3B*, *APOBEC3C*, *APOBEC3D*, *AICDA*. The dbSNP identifiers for the associated SNPs are: rs1014971. APOBEC/AID family is predicted as active if more than 5% of somatic mutations are attributed to its mutational signature. Deletion of *APOBEC3B* is determined by comparing read coverage of *APOBEC3B* versus the average coverage of its neighboring genes *APOBEC3A* and *APOBEC3C*. *APOBEC3B* is predicted to be homozygously deleted if its relative coverage falls below 0.25, heterozygously deleted if its relative coverage falls below 0.75, or otherwise not deleted.

RNA Fusion Analysis

Version 1.1

Using transcriptome-aligned RNA sequencing data, evidence of potential fusions is identified using clusters of spanning reads between two transcripts where one of the transcripts belongs to a gene among the following 74 genes commonly found in oncogenic fusions: ABL1, ABL2, AKT3, ALK, ARHGAP26, AXL, BCL2, BCOR, BCR, BRAF, BRCA1, BRCA2, BRD3, BRD4, CRLF2, EGFR, EPOR, ERG, ESR1, ETV1, ETV4, ETV5, ETV6, EWSR1, FGFR1, FGFR2, FGFR3, FGR, FOXO1, INSR, JAK2, KIT, KMT2A, MAML2, MAST1, MAST2, MEF2D, MET, MSH2, MSMB, MUSK, MYB, MYC, NOTCH1, NOTCH2, NRG1, NTRK1, NTRK2, NTRK3, NUMBL, NUTM1, PAX5, PDGFB, PDGFRA, PDGFRB, PIK3CA, PKN1, PPARG, PRKCA, PRKCB, RAF1, RARA, RELA, RET, ROS1, RSPO2, RSPO3, TCF3, TERT, TFE3, TFEB, THADA, TMPRSS2, ZNF384. The intragenic fusions ' known as EGFRvIII and MET exon 14 skipping are also identified by this method. When there is significant spanning read support, de novo assembly is performed on all sequencing data surrounding the approximate locations of the fusion in both genes to determine the precise location of the fusion within the genes' transcripts. When a precise location can be determined, the fusion transcript is generated and scanned for maximal open reading frame beginning at the start codon of the fusion's upstream partner. Only fusions that have open reading frames extending to the downstream partner's stop codon are considered as functional fusions and reported here.

For all functional fusions identified by the above method, evidence of structural variants in the DNA sequencing data of tumor and matched-normal (if available) samples is collected. The likely location of the fusion in the genome is determined by using the upstream and downstream partner's gene definitions and the location of the fusion event within those transcripts. The raw sequencing data in the DNA sequencing data are searched for evidence of reads spanning between the likely genomic locations.

Junction Analysis

Version 1.0

The raw RNA sequencing data is scanned for any support of junction sequences specific to the

following 3 gene fusions and splicing variants: AR-V7, EGFRvIII, MET Exon 14 Skip.

Structural variants

Version 1.2

Structural variants were identified using methods described in two publications on structural variation present in *Glioblastoma multiforme* tumors [31, 32]. The method works in two stages. The first stage identifies clusters of discordantly mapped reads (i.e. paired reads that map in an unexpected location and/or orientation, according to the reference human genome), identifying the approximate locations in the genome where a structural variant might exist. The second stage then searches the area around both ends of the structural variant to identify "split reads" that span the two sides of the structural variant. If split reads are discovered, this provides orthogonal evidence that the structural variant may be real while also refining the locations of the structural variant down to base-level precision. Such variants are considered "Precise." Structural variants found in regions of the human genome known to be difficult to accurately align, such as highly repetitive regions, are filtered from the analysis.

Structural variants are required to have a minimum support of at least 6 reads with an average mapping quality greater than 30. Variants that pass these criteria are then ranked according to the following heuristics (higher ranks: +2 if read support > 15, +4 if split read solution was found, +5 if it could create plausible fusion gene (i.e. correct orientation and phase), +4 for a "Near fusion" (i.e. correct orientation, but improper phase), +2 for deletion-type structural variants to could cause a loss of any part of a gene, +1 if variant interrupts a gene. Ranked structural variants that affect one or more cancer genes are highlighted in the report, while the full findings can be found in the supplemental files.

Provenance

Version 1.3

The germline genotypes are compared between tumor and matched-normal samples to determine if the samples belong to the same person. Up to 1,000 dbSNP loci are analyzed, and the percentage of sites that share identical or compatible genotypes between samples are used to determine similarity. An incompatible genotype occurs when the tumor sample is heterozygous, while the matched-normal sample is homozygous. Normal DNA samples with less than 3% incompatibility with Tumor DNA and less than 5% incompatibility with RNA (when available) are considered to be from the same person.

Homozygosity of X

Version 1.0

The germline genotypes for all single nucleotide dbSNP loci on chromosome X are used to calculate the homozygosity percentage for each sample presented in this report. For the purposes of this analysis, a locus is deemed homozygous in a sample if the maximum allele fraction (reference or alternate allele) exceeds 0.65. Only loci with read depths greater than or equal to 10 reads across all samples are considered. If more than 75% of all chromosome X loci meeting this criteria are homozygous, the sample is likely male in origin, barring any copy number alterations present on chromosome X.

Contrast Summary

Version 1.0

The human genome reference build 37 (hg19) was used to align and analyze all sequencing data produced for this report. All SNV and small indel variants were annotated against common polymorphisms (found in at least 1% of the population) from dbSNP build 138.

Sequence Information

Version 1.7

The human genome reference build 37 (hg19) was used to align and analyze all sequencing data produced for this report. All SNV and small indel variants were annotated against common polymorphisms (found in at least 1% of the population) from dbSNP build 142.

Throughout this report, gene names are colored according to their gene class, where oncogenes are colored *RED*, tumor suppressors are colored *BLUE*, oncogene/tumor suppressors are colored *PURPLE*, and other genes are colored black. Gene classes are obtained using data from COSMIC Cancer Gene Census [1].

For each aligned sample, the numbers of total reads, mapped, and duplicates are collected. Average coverage is estimated for each exon (coding and non-coding), intron, and intergenic region between genes. Coverage of the whole genome and exome are calculated by aggregating the coverage estimates for all regions and exome regions, respectively.

When these granular coverages estimates are unavailable, average coverage is calculated by taking the total number of mapped reads multiplied by read length and divided by the total number of bases in the human genome or exome, as appropriate. This does not take into account unalignable regions of the genome, so these estimates may underestimate the true coverage of the genome.

In addition, a variety of summary metrics is computed for each position within sequencing reads and shown in the set of figures in the appendix. These metrics can help identify problems with the input sequence. The base composition of the reads gives the percentage of reads with a particular base at a given position in the reads, which can identify the presence of an adapter sequence that should be clipped from the input sequence. Base quality is split into three bins: High if $q > 19$, Low if $q < 6$, or Average. In general, base quality worsens near the end of the sequencing reads, so do not be concerned if such a pattern is observed. Finally, the per-base alignment statistics are computed for the following alignment categories: deletion (D), insertion (I), skipped (N), soft clip (S), hard clip (H), and padding (P).

Sample Contamination

Version 1.0

All somatic small variants detected in the tumor sample are analyzed for how common they are in the global human population. Variants that are found in more than 5.0% of the population are considered *common*. The number of common sites versus the total number of somatic small variants detected is used to determine if the tumor sample is contaminated with the DNA of an unrelated individual.

In addition, somatic variants present in the matched-normal are tallied to determine if DNA from the tumor sample has potentially contaminated the matched-normal sample. High levels of such contamination can reduce sensitivity of somatic variant detection. A somatic variant is classified as *present* in the matched-normal sequencing data if the variant allele is found in more than 5.0% of the total reads at that site.

RNA Quality

Version 1.0

Gene- and exon-level coverages are computed by calculating the average read depth across all coding exons defined by the canonical isoform of the gene.

Expression status of genes with mutant alleles is determind using the lower and upper 5th percentiles of per-gene RSEM TPM values from a collection of RNA sequencing datasets from TCGA normal samples. The expression status for a gene is classified as "Over-expressed" if its TPM exceeds the gene's upper 5th percentile, "Under-expressed" if it is less than the lower 5th percentile, "Not expressed" if its TPM value equals zero, or otherwise classified as having a "Normal" expression status. If a gene's upper or lower 5th percentile is unavailable, the expression status for that gene is classified as "N/A".

About This Test

Version 1.1

The following paragraph provides references to the published methods employed in preparation of the DNA and RNA sequencing data prior to analysis by the NantOmics Contraster analysis pipeline.

DNA sequencing data is aligned to GRCh37 (www.ncbi.nlm.nih.gov/assembly/2758/) by bwa [34]. Duplicates marked by `samblaster` [35]. Indel realignment and base quality recalibration performed by `GATK v2.3` [36]. RNA sequencing data aligned by `bowtie` [37]. RNA transcript expression estimated by `RSEM` [25].

References

[1] Forbes, S. et al. *COSMIC: exploring the world's knowledge of somatic mutations in human cancer*. **Nucl. Acids Res.**, 43:D805-D811, October 2014.

[2] Rizni, N.A. et al. *Cancer immunology. Mutational landscape determines sensitivity to PD-1 blockade in non-small cell lung cancer.*. **Science**, 348(6230):124-128, April 2015.

[3] Alshehri, A. et al. *Protein signature defines non-squamous NSCLC responders to pemetrexed therapy*. **IASLC Annual Meeting**, September 2015.

[4] Nuciforo, P. et al. *High Levels of HER2 Measured By Multiplex Mass Spectrometry Correlate With Increased Overall Survival In Patients Treated With Anti-HER2 Therapy*. **ASCO Annual Meeting**, June 2014.

[5] Nuciforo, P. et al. *High HER2 protein levels correlate with increased survival in breast cancer patients treated with anti-HER2 therapy*. **Molecular Oncology (In Press)**, September 2015.

[6] Hembrough, T. et al. *Quantification of HER2 from gastroesophageal cancer (GEC) FFPE tissue by mass spectrometry (MS)*. **Gastrointestinal Cancers Symposium**, January 2014.

[7] Liao, W-L et al. *Development of a Multiplexed Mass Spectrometry Assay to Differentiate Adeno/Squamous Carcinoma in Non-Small Cell Lung Cancer*. **ASMS Annual Meeting**, June 2012.

[8] Catenacci, D. et al. *Absolute quantification of Met using mass spectrometry for clinical application: Assay precision, stability, and correlation with MET gene amplification in FFPE tumor tissue*. **PLoS One**, 9(7):e100586, July 2014.

[9] Carithers, L.J. et al. *A Novel Approach to High-Quality Postmortem Tissue Procurement: The GTEx Project*. **Biopreserv Biobank**, October 2015.

[10] Weinstein, J.N. et al. *The Cancer Genome Atlas Pan-Cancer analysis project*. **Nature Genetics**, September 2013.

[11] Moore, B. et al. *VAAST 2.0: Improved Variant Classification and Disease-Gene Identification Using a Conservation-Controlled Amino Acid Substitution Matrix*. **Genet Epidemiol**, 37:622-634, May 2013.

[12] Tamborero, D. et al. *Comprehensive identification of mutational cancer driver genes across 12 tumor types*. **Scientific Reports**, 3:2650, October 2013.

[13] Tamborero, D. et al. *OncodriveCLUST: exploiting the positional clustering of somatic mutations to identify cancer genes*. **Bioinformatics**, 7:1-7, July 2013.

[14] Landau, D. et al. *Evolution and Impact of Subclonal Mutations in Chronic Lymphocytic Leukemia*. **Cell**, 152:714-726, February 2013.

[15] Kalia, S.S. et al. *Recommendations for reporting of secondary findings in clinical exome and genome sequencing, 2016 update (ACMG SF v2.0): a policy statement of the American College of Medical Genetics and Genomics*. **Genetics in Medicine**, 19(2):249-255, October 2017.

[16] Szabo, C. et al. *The Breast Cancer Information Core: Database design, structure, and scope*. **Human Mutation**, 16(2):123-131, July 2000.

[17] Salipante, S.J. et al. *Microsatellite Instability Detection by Next Generation Sequencing*. **Clinical Chemistry**, February 2014.

[18] Vivian, J. et al.. *Rapid and efficient analysis of 20,000 RNA-seq samples with Toil*. **bioRXiv [Epub ahead of print] doi:10.1101/062497**, 2016.

[19] Cortes, C. et al.. *Support-vector networks*. **Machine Learning**, 1995.

[20] Guyon, I. et al.. *Automatic Capacity Tuning of Very Large VC-dimension Classifiers*. **Advances in Neural Information Processing**, 1993.

[21] van der Maaten, L.J.P. et al.. *Visualizing High-Dimensional Data Using t-SNE*. **Journal of Machine Learning Research**, 2008.

[22] van der Maaten, L.J.P.. *Learning a Parametric Embedding by Preserving Local Structure*. **Proceedings of the Twelfth International Conference on Artificial Intelligence & Statistics**, 2009.

[23] Perou, CM et. al. *Molecular portraits of human breast tumours*. **Nature**, August 2015.

[24] Guinney et al. *The Consensus Molecular Subtypes of Colorectal Cancer*. **Nature Medicine**, November 2015.

[25] Li, Bo and Dewey, Colin N.. *RSEM: accurate transcript quantification from RNA-Seq data with or without a reference genome*. **BMC Bioinformatics**, August 2011.

[26] Pardoll, D.M.. *The blockade of immune checkpoints in cancer immunotherapy*. **Nature Reviews Cancer**, 12:252-264, April 2012.

[27] Rizvi, N.A. and Chan, T.A.. *Mutational landscape determines sensitivity to PD-1 blockade in non-small cell lung cancer*. **Science**, 348:124-128, April 2015.

[28] Platten, M. et al. *Cancer immunotherapy by targeting IDO1/TDO and their downstream effectors*. **Frontier in Immunology**, 5:673-674, January 2015.

[29] Anderson, A.C.. *Tim-3: An Emerging Target in the Cancer Immunotherapy Landscape*. **Cancer Immunology Research**, 2:393-398, May 2014.

[30] Bindea, G. et al.. *Spatiotemporal dynamics of intratumoral immune cells reveal the immune landscape in human cancer.*. **Immunity**, October 2013.

[31] Sanborn, J. Zachary et al.. *Double Minute Chromosomes in Glioblastoma Multiforme Are Revealed by Precise Reconstruction of Oncogenic Amplicons*. **Cancer Res**, 73(19):1-10, September 2013.

[32] Brennan, Cameron W. et al.. *The Somatic Genomic Landscape of Glioblastoma*. **Cell**, 155(2):462-477, October 2013.

[33] Alexandrov LB et al.. *Signatures of mutational processes in human cancer*. **Nature**, 500(7463):415-421, August 2013.

[34] Li, H. et al. *Fast and accurate short read alignment with Burrows-Wheeler transform*. **Bioinformatics**, 25(14):1754-1760, February 2009.

[35] Faust, G.G. et al. *SAMBLASTER: fast duplicate marking and structural variant read extraction*. **Bioinformatics**, 30(17):2503-2505, September 2014.

[36] DePristo, M. et al. *A framework for variation discovery and genotyping using next-generation DNA sequencing*. **Nature Genetics**, 43:491-498, 2011.

[37] Langmead, B. et al. *Ultrafast and memory-efficient alignment of short DNA sequences to the human genome*. **Genome Biology**, 10(3):25, 2009.

Disclaimer

This report is for informational purposes only, and is not intended to diagnose or treat any disease. The treatment of patients with any agents mentioned in this report resides solely with the discretion of the treating physician. The mere presence of genomic alterations in genes that are targeted by agents does not indicate sensitivity to that particular agent. The accuracy of this report is based solely on the data provided, which may contain errors obtained during sequencing or other downstream analysis. Any findings here should be verified with a qualified test in a CLIA laboratory setting.

Appendix

Descriptions of Curated Findings biomarkers detected in sample

Biomarker Description

MSI-H

MSI-H High-frequency microsatellite instability is characterized by hypermutability within microsatelite DNA, which consists of tandemly repeated DNA motifs located throughout regulatory, coding and non-coding DNA.

high MGMT expression vs. low MGMT expression

MGMT gene encodes a DNA methyltransferase involved in DNA repair. Provides cellular defense from mutagenic DNA alkylating agents. Catalyzes the removal of methyl moeities from O6-methylguanine and O4-methylthymine.

expresses TOP1

expresses TOP1 Topoisomerase 1, DNA topoisomerase that regulates DNA topology by catalyzing single-strand breaks, involved in DNA replication, recombination, transcription and repair.

KRAS G12D

KRAS G12D mutation encodes a missense substitution that abrogates GTPase-activating protein (GAP)-dependent KRAS inactivation, resulting in constitutive KRAS GTPase activity.

KRAS mutation in codon 12 or 13

Mutations at codon position 12 or 13 (within exon 2) are oncogenic activating mutations that have been observed across numerous tumor types, and are associated with resistance to some EGFR inhibitor therapies. KRAS gene encodes a small membrane-associated GTPase involved in the regulation of PI3K and RAF/MEK/ERK pathway signaling.

KRAS G12X

KRAS G12X mutations are one of the most frequently occuring missense mutations in KRAS, observed in up to 95% of all pancreatic cancers, but also identified across numerous cancer types. Substitutions at position G12 are oncogenic activating mutations and are associated with resistance to some EGFR inhibitor therapies.

KRAS mutation in codons 12, 13, 59, 61, 117 or 146

KRAS mutation in codons 12, 13, 59, 61, 117 or 146 are observed across numerous cancer types. KRAS gene encodes a small membrane-associated GTPase involved in the regulation of PI3K and RAF/MEK/ERK pathway signaling.

Remaining Pathogenic, Likely Pathogenic, or Unknown Significance mutations ranked by pathogenicity

Category	Gene	Class	Variant	Alt. / Total	RNA Alt. / Total	Details
Unkn. Sig.	*PTPRS*	Nonsense	`p.R235*`	5 / 113	6 / 177	RNA+, NMD, Subclonal
Unkn. Sig.	*ABCA7*	Nonsense	`p.C1018*`	5 / 117	6 / 160	RNA+, NMD, Subclonal
Unkn. Sig.	*LEPR*	Missense	`p.F898L`	5 / 41	1412 / 1454	RNA+, Driver Gene, Subclonal
Unkn. Sig.	*LEPR*	Missense	`p.H924P`	5 / 46	7474 / 7943	RNA+, Driver Gene, Subclonal
Unkn. Sig.	*DYNC1I1*	Frame Shift	`p.G579Rfs*42`	5 / 84	507 / 2417	RNA+, Not NMD, Subclonal
Unkn. Sig.	*TJP1*	Missense	`p.L1238I`	5 / 125	0 / 293	RNA-, CASM High, Subclonal
Unkn. Sig.	*SCYL2*	Nonsense	`p.K665*`	5 / 27	21 / 143	RNA+, NMD
Unkn. Sig.	*GUCY2C*	Nonsense	`p.R497*`	4 / 46	12 / 277	RNA+, NMD, Subclonal
Unkn. Sig.	*KDM3B*	Missense	`p.R1046H`	4 / 60	34 / 421	RNA+, Putative Tumor Suppressor, Subclonal
Unkn. Sig.	*DVL3*	Frame Shift	`p.H632Qfs*36`	4 / 65	21 / 351	RNA+, Not NMD, Subclonal
Unkn. Sig.	*GABRA6*	Frame Shift	`p.V435Cfs*4`	4 / 23	0 / 0	RNA-, Not NMD
Unkn. Sig.	*AGBL4*	Nonsense	`p.Y135*`	4 / 23	6 / 12	RNA+, NMD, Subclonal
Unkn. Sig.	*NKAPL*	Frame Shift	`p.K239Rfs*39`	3 / 21	2 / 22	RNA+, Not NMD, Subclonal
Unkn. Sig.	*SERPINB4*	Frame Shift	`p.W201Gfs*12`	3 / 15	0 / 0	RNA-, NMD

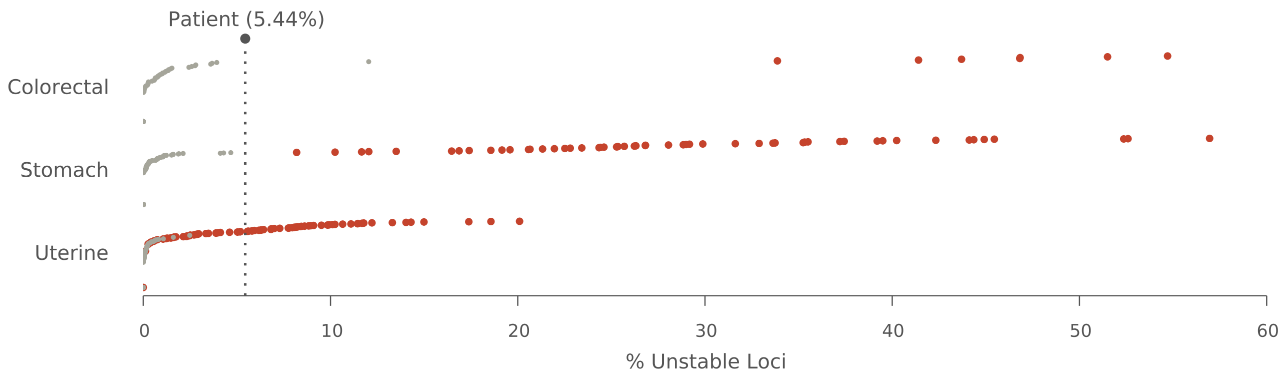

Figure 1: Comparison of patient tumor's estimated microsatellite instability versus TCGA tumors with known MSI status. Red markers denote TCGA tumors known to have high microsatellite instability.

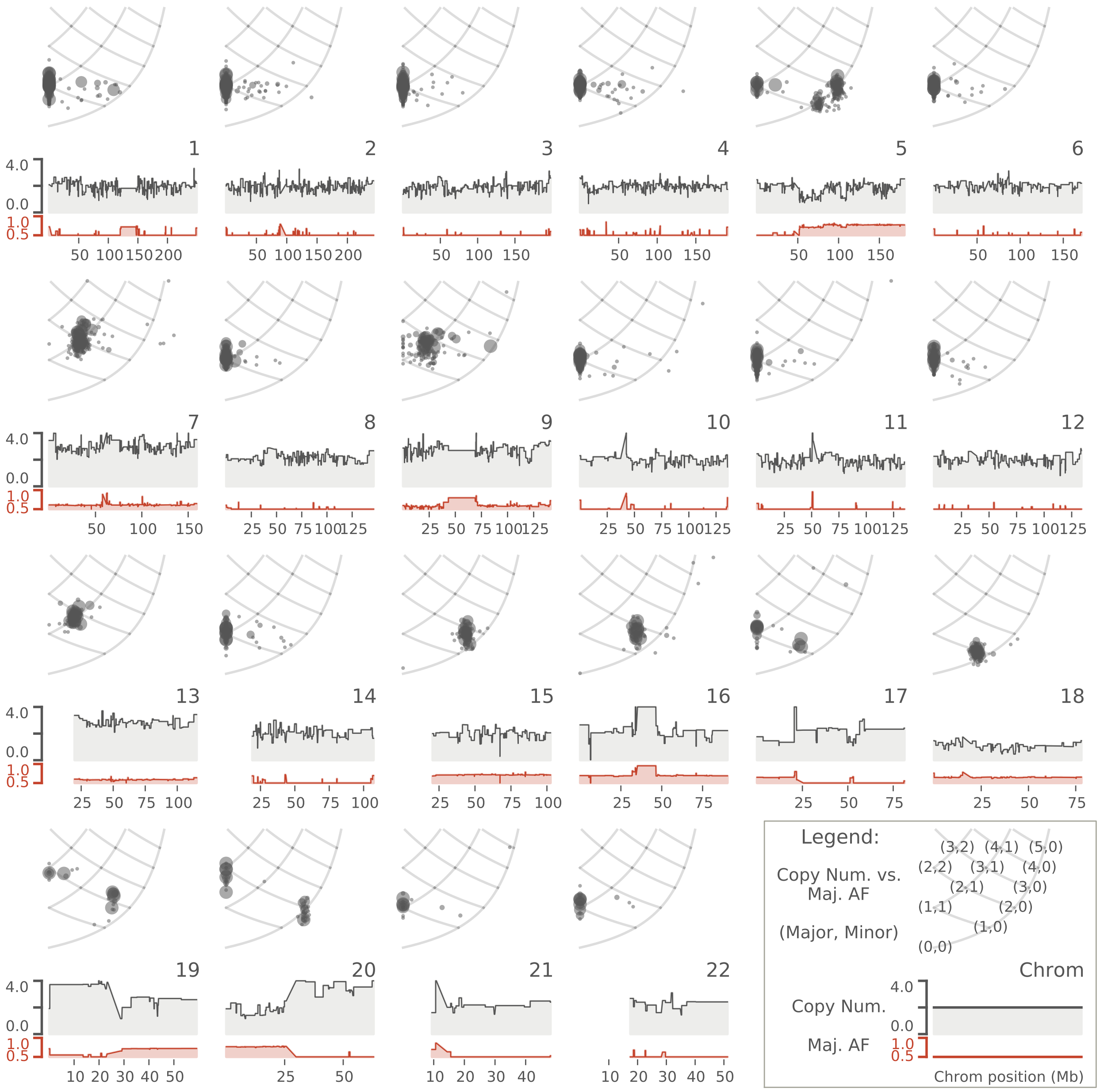

Figure 2: Allelic state diagrams and chromosomal plots for the 22 autosomes according to an estimated 45% normal contamination. Each copy number segment is represented on both the ASD (as a circle with radius scaled by the segment's size) and below in the chromosomal plot. A legend that helps explain the layout of each diagram can be found in the bottom right of the figure. Maj. AF = majority allele fraction, Major = Majority allele copy number, Minor = Minority allele copy number.

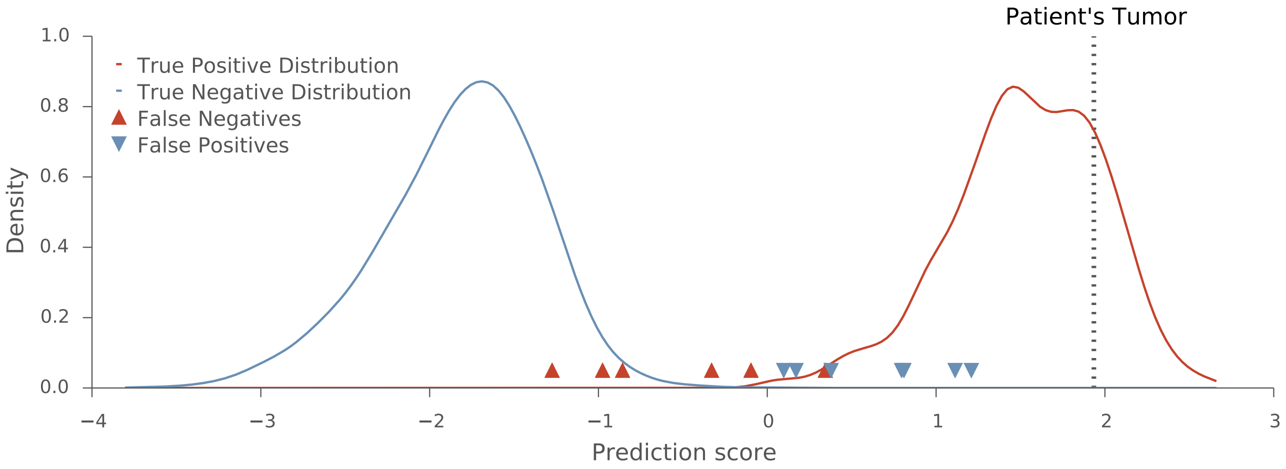

Figure 3: Density plot of all scores for validation samples predicted to be **Colorectal** by the site of origin prediction model. The score for the patient's tumor is indicated by the dotted line.

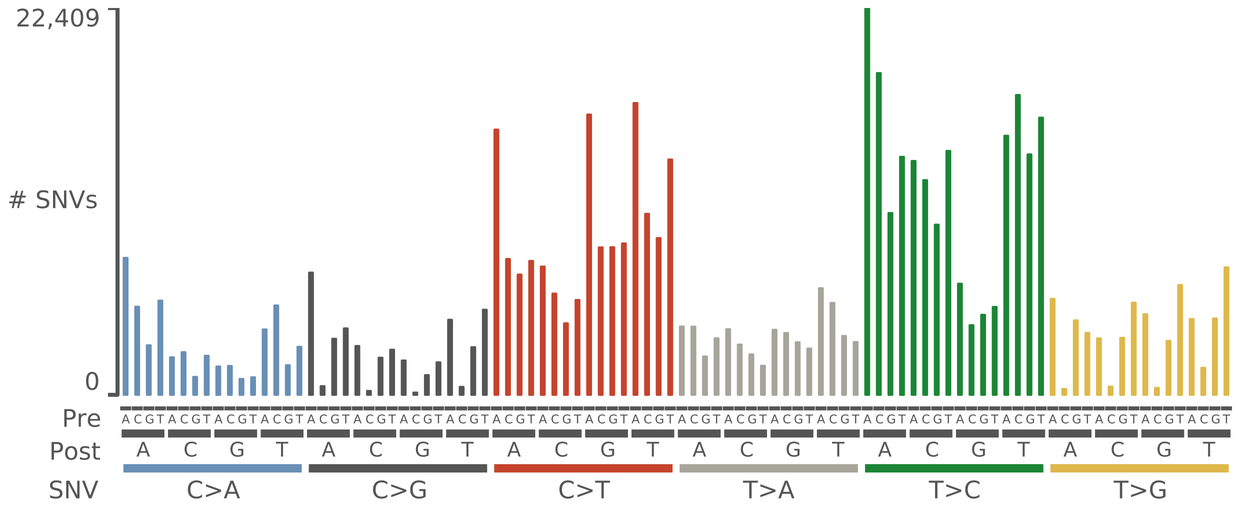

Figure 4: Plot of mutation counts by their genomic contexts

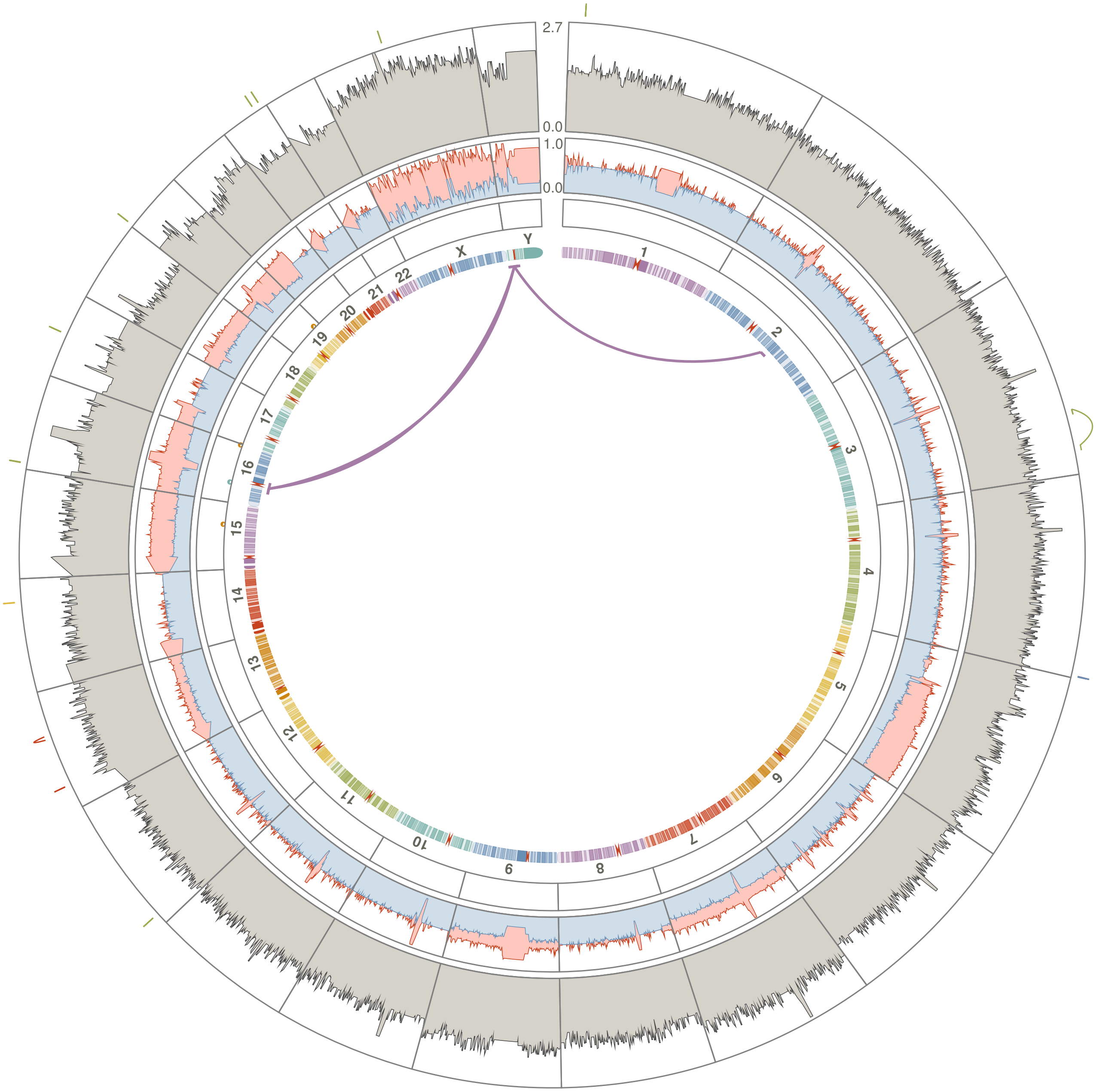

Figure 5: Circular genome plot. Moving inwards from the outside: intra-chromosomal structural variants (lines), overall copy number (gray), majority and minority allele fractions (red and blue, respectively), SNVs and small indels (colored dots), and inter-chromosomal structural variants. Structural variants drawn with thick colored lines were found to have supporting split reads.

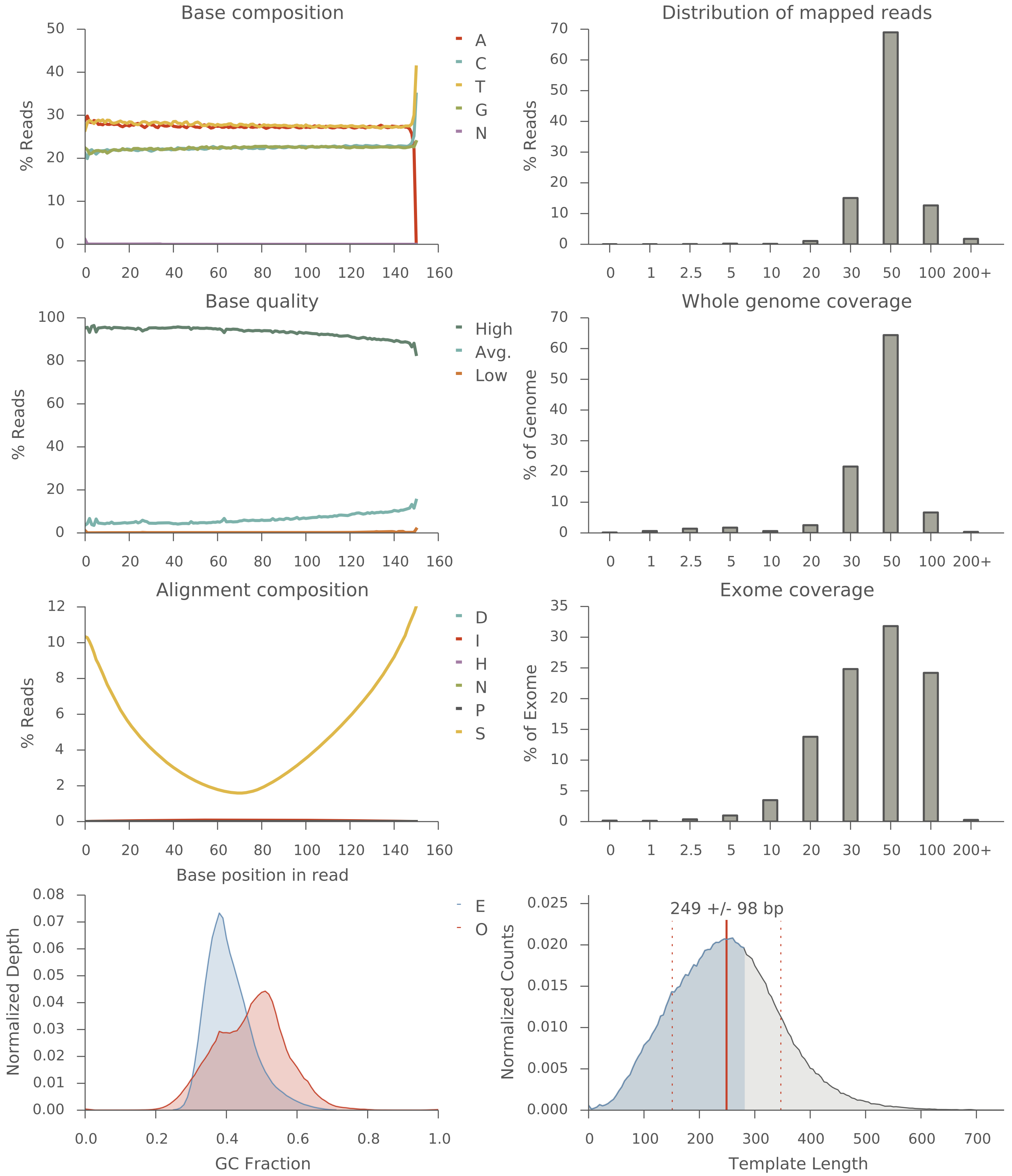

Figure 6: Sample quality metrics for Tumor WGS. Key for legends: D = Deletion, I = Insertion, N = Skipped, H = Hard clip, S = Soft clip, P = Padding, E = Expected GC distribution, and O = Observed GC distribution. Blue shaded area in the Template Length figure indicates portion of the distribution with overlapping read pairs.

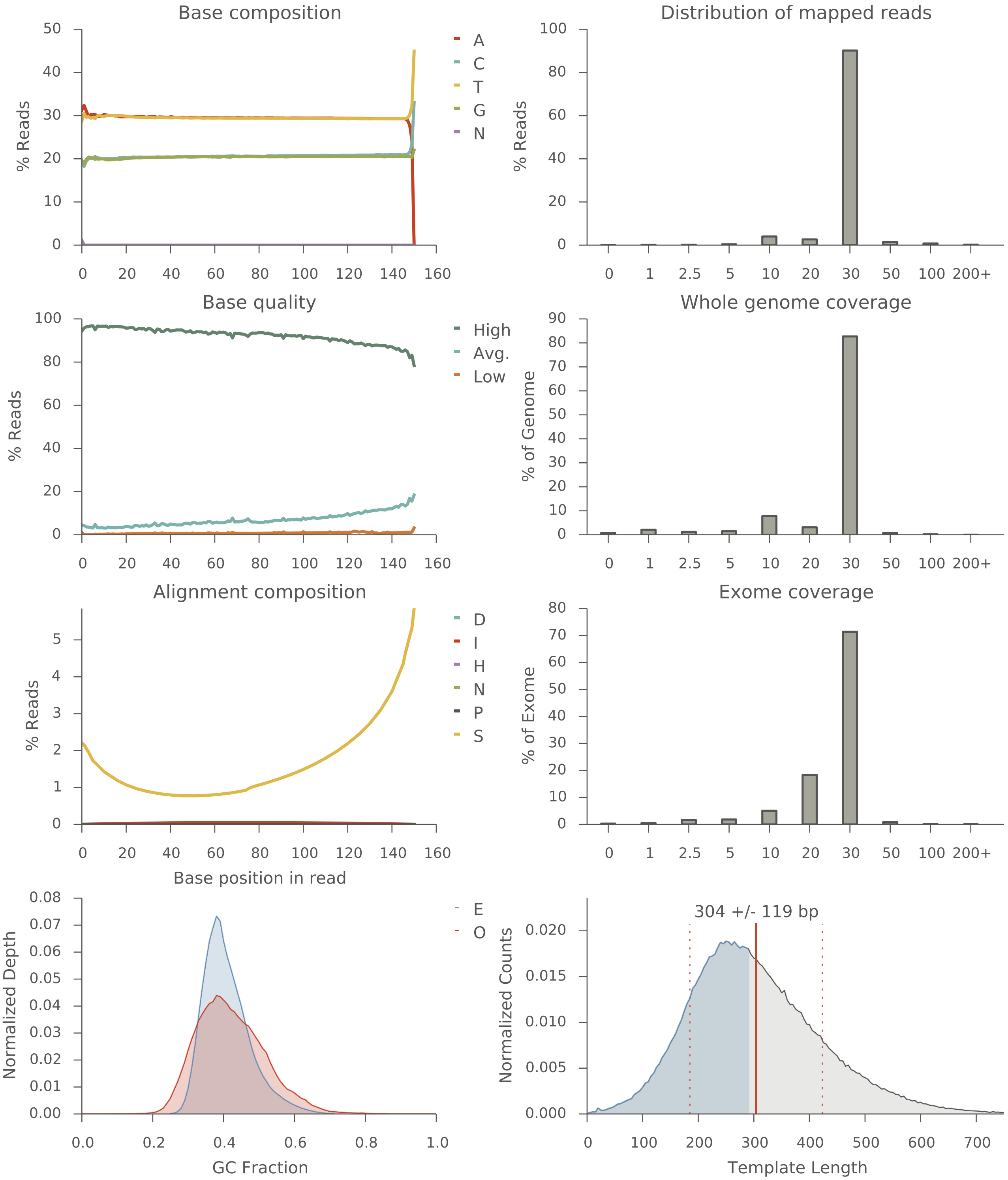

Figure 7: Sample quality metrics for Normal WGS. Key for legends: D = Deletion, I = Insertion, N = Skipped, H = Hard clip, S = Soft clip, P = Padding, E = Expected GC distribution, and O = Observed GC distribution. Blue shaded area in the Template Length figure indicates portion of the distribution with overlapping read pairs.

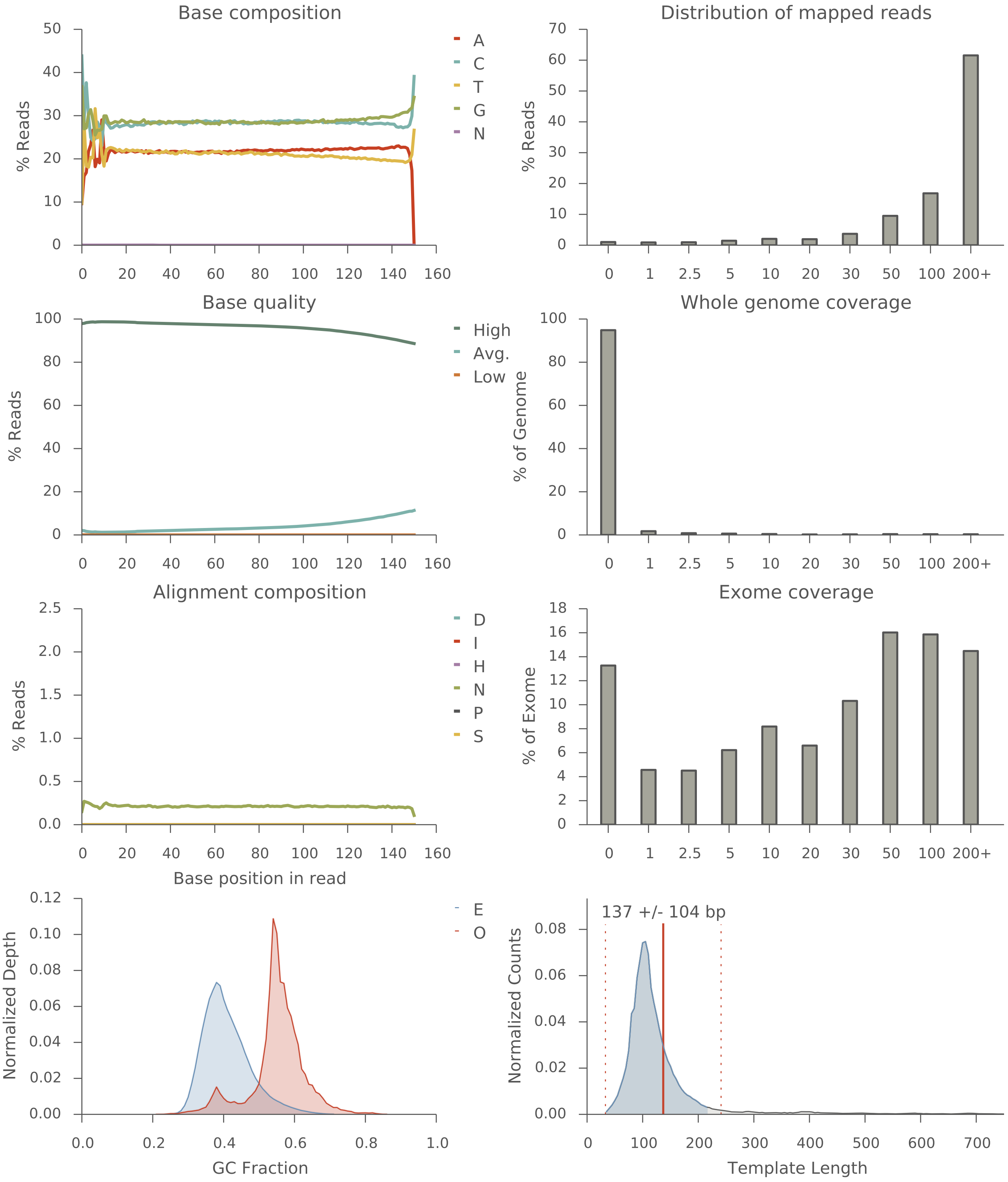

Figure 8: Sample quality metrics for Tumor RNA. Key for legends: D = Deletion, I = Insertion, N = Skipped, H = Hard clip, S = Soft clip, P = Padding, E = Expected GC distribution, and O = Observed GC distribution. Blue shaded area in the Template Length figure indicates portion of the distribution with overlapping read pairs.

Glossary of Terms

Allele Fraction (AF) Expressed as a percentage of reads supporting the variant allele. When tumor purity and ploidy measurements are available, allele fraction is corrected for tumor purity to more accurately reflect the allele fraction of the variant allele in tumor cells. (*Genomics, Transcriptomics*)

Alt. / Total Number of DNA (or RNA) reads supporting the variant allele vs. the the total number of reads mapped to the location of variant allele. (*Genomics, Transcriptomics*)

Biomarker A biological marker that is relevant from a clinical or cancer research standpoint. These markers can be RNA expression levels, somatic or germline small variants, gene fusions, or copy number alterations. (*Genomics, Transcriptomics*)

CASM Conservation-controlled Amino acid Substitution Matrix. Estimates how disruptive a particular amino acid change is to the gene's function. (*Genomics*)

DNA+ Support for variant in DNA sequencing data of tumor sample (and matched-normal sample if variant is germline). (*Genomics*)

DNA- Lack of support for variant in DNA sequencing data of tumor sample. (*Genomics*)

Exon The region of the genome belonging to part of the coding sequence of a gene. The sequence of all exonic regions for a given gene encodes its protein. (*Genomics*)

Frame Shift An insertion or deletion within the coding region of gene that shifts the translation reading frame of the protein and usually results in the introduction of a premature stop codon. (*Genomics*)

In-Frame Del./Ins. An insertion or deletion within the coding region of gene that alters the protein but does not shift the translation reading frame. (*Genomics*)

Intergenic The region of the genome found in between genes. (*Genomics*)

Intron The region of the genome found between exons of a gene. (*Genomics*)

Missense A single nucleotide mutation that results in a change to the amino acid sequence of the protein, but does not introduce a premature stop codon. (*Genomics*)

NMD Nonsense-Mediated mRNA Decay. A surveillance pathway to reduce transcription errors that is triggered when a premature stop codon is encountered 50bp prior to the start of the gene's last exon. (*Genomics*)

Nonsense A single nucleotide mutation that introduces a premature stop codon to the protein. Depending on the location of the premature stop codon within the protein sequence, nonsense-mediated decay of the protein product may or may not be triggered. (*Genomics*)

RNA+ Support for variant in RNA sequencing data of tumor sample. For small variants, this requires a minimum of 2 RNA reads supporting the variant allele. (*Transcriptomics*)

RNA- Lack of support for variant in RNA sequencing data of tumor sample. (*Transcriptomics*)

Silent A single nucleotide mutation that results in no change to the amino acid sequence of the protein. (*Genomics*)

TPM Transcripts Per kilobase Million (TPM) is a measurement of gene (or gene isoform) expression determined using RNA sequencing data. (*Transcriptomics*)
